# Metabolic mediators: microbial-derived metabolites as key regulators of anti-tumor immunity, immunotherapy, and chemotherapy

**DOI:** 10.3389/fimmu.2024.1456030

**Published:** 2024-09-16

**Authors:** Shan Lu, Chunling Wang, Jingru Ma, Yichao Wang

**Affiliations:** ^1^ Department of General Practice, The Second Hospital of Jilin University, Changchun, China; ^2^ Medical Affairs Department, The Second Hospital of Jilin University, Changchun, China; ^3^ Department of Clinical Laboratory, the Second Hospital of Jilin University, Changchun, China; ^4^ Department of Obstetrics and Gynecology, the Second Hospital of Jilin University, Changchun, China

**Keywords:** microbial-derived metabolites, anti-tumor immunity, chemotherapy, immunotherapy, therapeutic strategies

## Abstract

The human microbiome has recently emerged as a focal point in cancer research, specifically in anti-tumor immunity, immunotherapy, and chemotherapy. This review explores microbial-derived metabolites, emphasizing their crucial roles in shaping fundamental aspects of cancer treatment. Metabolites such as short-chain fatty acids (SCFAs), Trimethylamine N-Oxide (TMAO), and Tryptophan Metabolites take the spotlight, underscoring their diverse origins and functions and their profound impact on the host immune system. The focus is on SCFAs’ remarkable ability to modulate immune responses, reduce inflammation, and enhance anti-tumor immunity within the intricate tumor microenvironment (TME). The review critically evaluates TMAO, intricately tied to dietary choices and gut microbiota composition, assessing its implications for cancer susceptibility, progression, and immunosuppression. Additionally, the involvement of tryptophan and other amino acid metabolites in shaping immune responses is discussed, highlighting their influence on immune checkpoints, immunosuppression, and immunotherapy effectiveness. The examination extends to their dynamic interaction with chemotherapy, emphasizing the potential of microbial-derived metabolites to alter treatment protocols and optimize outcomes for cancer patients. A comprehensive understanding of their role in cancer therapy is attained by exploring their impacts on drug metabolism, therapeutic responses, and resistance development. In conclusion, this review underscores the pivotal contributions of microbial-derived metabolites in regulating anti-tumor immunity, immunotherapy responses, and chemotherapy outcomes. By illuminating the intricate interactions between these metabolites and cancer therapy, the article enhances our understanding of cancer biology, paving the way for the development of more effective treatment options in the ongoing battle against cancer.

## 1 Introduction

The mammalian gut constitutes a highly intricate ecosystem comprised of billions of mutually beneficial microorganisms, encompassing archaea, bacteria, protists, fungi, and viruses. Notably, bacteria are particularly abundant in the gut (1). The gut microbiota has been extensively studied and is important in a variety of physiological activities, including immune system development and the generation of critical nutrients (2–4). Its importance extends to disease prevention, influencing human health through the production of essential metabolites, nutrition metabolism, and the detoxification of hazardous substances. These actions inhibit the proliferation of detrimental microorganisms and contribute to the production of beneficial microbial products. Moreover, the microbiota plays a crucial role in metabolizing nutrients and toxins introduced by invading species (5). Microorganisms produce three main classes of metabolites: those derived from exogenous substances, those synthesized by the host and chemically modified by gut bacteria, and those generated from scratch by gut microorganisms (6). About 10% of metabolites in mammalian blood originate from the gut microbiota. These metabolites can exert diverse effects on the immune system and the overall equilibrium of the host’s body by binding to specific receptors and triggering subsequent signaling cascades (7). SCFAs (e.g., butyrate, acetate, and propionate) (8–10), tryptophan metabolites (e.g., indole, indole-3-acetic acid (IAA), 3-indole acrylic acid (IA), indole-3-aldehyde (IAld), indole-3-lactic acid (ILA), and tryptamine (11), and TMAO (12) are among the extensively researched microbial metabolites that are associated with human health and diseases. Recent research indicates that certain gut bacteria species and their byproducts may play a role in cancer immunity, immunotherapy, and chemotherapy (13).

The term “anti-tumor immunity” refers to the innate and adaptive immune responses that lead to the suppression of tumors (14). Dendritic cells (DCs) capture and degrade antigens within tumors. Activated DCs migrate to nearby lymph nodes to activate anti-tumor T cells; these T cells infiltrate tumors and are reactivated in the tissue to destroy the tumor cells and finally start cleaning. Destroying tumor cells while restoring normal tissue structure and immune system balance are critical steps in an effective immune response against tumors (15). The composition of the gut microbiota has a profound impact on the body’s immune responses against tumors, influencing the effectiveness of cancer immunotherapy, particularly treatments involving immune checkpoint inhibitors (ICIs). Specific beneficial or harmful bacterial species have been identified to either enhance or impair the immune response against tumors in various types of cancer (16–19). Recent studies emphasize the critical role of metabolites produced by the gut microbiota in determining the outcomes of anti-tumor therapies (20–23). For instance, Zhang et al. conducted a study to investigate the effects of a particular probiotic strain, Lactobacillus plantarum L168, and its metabolite, ILA, on colorectal cancer (CRC) using a mouse model (24). They investigated fundamental mechanisms and determined that ILA played a crucial role. It was revealed that this metabolite enhances the production of interleukin-12 subunit alpha (IL12a) in DCs (24). This acceleration was achieved by increasing the binding of H3K27ac to the enhancer regions of IL12a, facilitating the initiation of a CD8+ T cell immune response, particularly targeting tumor proliferation.

**Figure 1:**
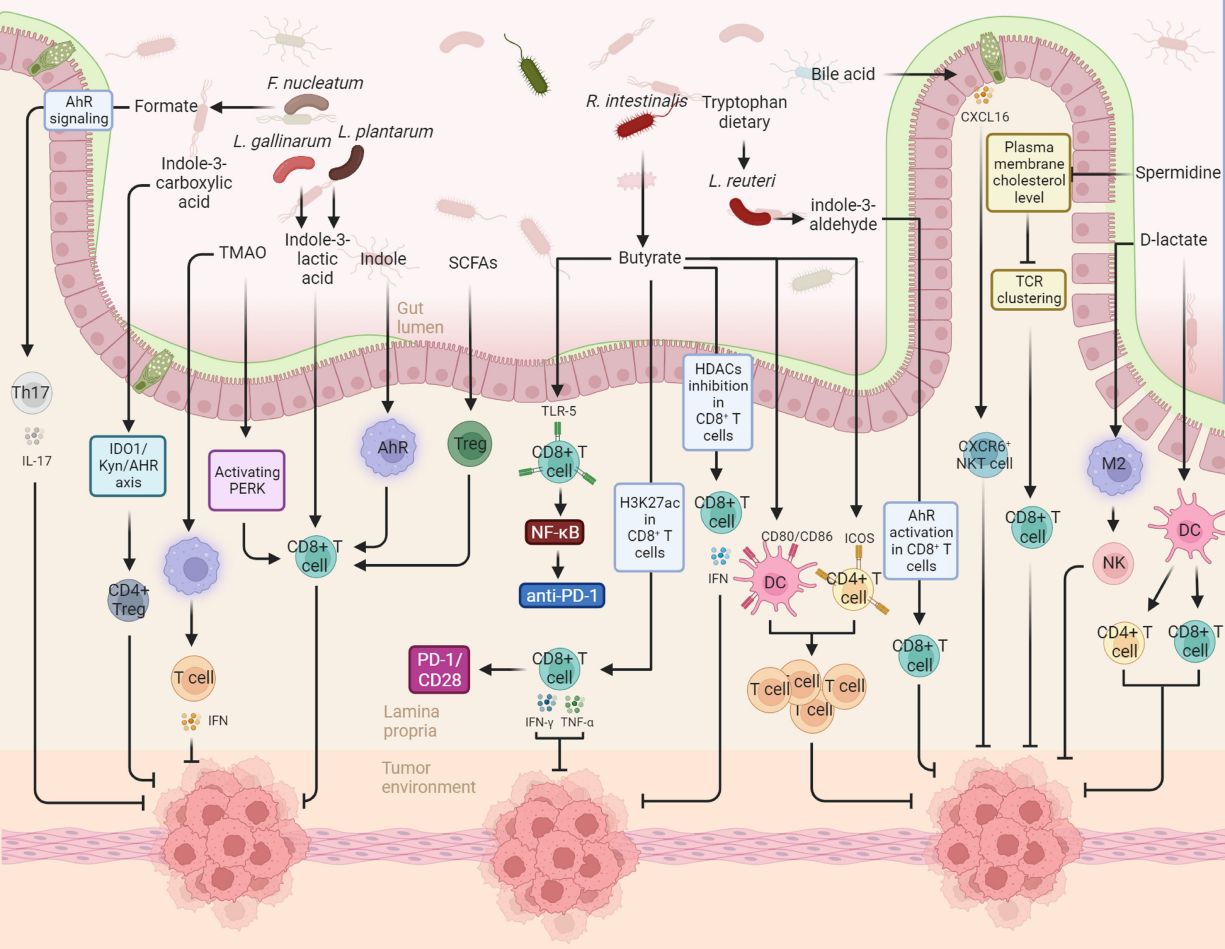
This figure illustrates the diverse actions and underlying mechanisms of gut microbiota-derived metabolites in modulating the immune landscape against cancer, specifically in the realms of anti-tumor immunity and cancer immunotherapy. For instance, in the context of anti-tumor immunity, gut-derived indole-3-lactic acid promotes antitumor responses through the epigenetic regulation of CD8+ T cell immunity. The production of indole-3-lactic acid by *Lactobacillus plantarum* is shown to enhance colorectal carcinogenesis by epigenetically regulating the immune response of CD8+ T cells. Conversely, microbial metabolites derived from tryptophan activate the aryl hydrocarbon receptor (AHR) in tumor-associated macrophages, resulting in the suppression of anti-tumor immunity. In the context of cancer immunotherapy, *Roseburia intestinalis*-produced butyrate is highlighted as an enhancer of anti-PD-1 treatment efficacy. This effect is attributed to butyrate’s stimulation of cytotoxic CD8+ T cells, achieved through its direct binding to toll-like receptor 5 (TLR5) on CD8+ T cells.

Cancer immunotherapy holds the potential to revolutionize cancer treatment by exploring and developing innovative methods that enhance the body’s innate capacity to combat tumors (25–27). Recent research underscores the significant role of metabolites originating from the gut microbiota in determining the efficacy of cancer immunotherapy (28–31). For instance, Kang et al. demonstrated that Roseburia intestinalis protects against CRC formation by producing butyrate, thereby enhancing the effectiveness of anti-Programmed death protein 1 (PD1) immunotherapy (28) (**Figure 1**, **Table 1**). Chemotherapy, the primary therapeutic approach for malignant tumors, relies on inducing cell death through the use of various medications, such as antimetabolites, alkylating agents, mitotic spindle inhibitors, antitumor antibiotics, and hormonal anticancer therapies (32). Chemotherapy generally functions by inhibiting the division and proliferation of cancer cells. Cancer cells, characterized by rapid division and growth compared to normal cells, experience high intrinsic physiological stress, making them susceptible to swift and efficient elimination by these medications (33). Moreover, chemotherapy exists in various forms, each exerting distinct effects on specific target cells (34). Recent studies highlight the crucial role of metabolites produced by gut bacteria in influencing the effectiveness of cancer treatment (35–37). Tintelnot et al. investigated the influence of nutrition on therapeutic responses with special emphasis on 3-IAA, a tryptophan metabolite produced by the microbiota (35). Their study revealed that patients exhibiting a positive response to therapy displayed elevated levels of 3-IAA, as identified through shotgun metagenomic sequencing and metabolomic screening (35). Furthermore, they highlighted the significant role of myeloperoxidase, derived from neutrophils, in influencing the efficacy of 3-IAA and chemotherapy. Myeloperoxidase catalyzes the oxidation of 3-IAA, leading to a reduction in the activity of enzymes responsible for breaking down reactive oxygen species (ROS). Consequently, this results in an accumulation of ROS levels and a concurrent decrease in autophagy within cancer cells. These alterations compromise the metabolic efficiency of cancer cells, ultimately impeding their growth (35). Besides, Colbert et al. found that tumor-resident *Lactobacillus iners* can induce chemoradiation resistance in cervical cancer through lactate-induced metabolic changes (36). This study employs deep microbiome sequencing, targeted bacterial culture, and *in vitro* assays to analyze how tumor and gut microbiota impact chemoradiation response. The findings reveal that *Lactobacillus iners*, an L-lactate-producing bacterium in tumors, is linked to decreased patient survival and induces resistance to chemotherapy and radiation by altering tumor metabolic pathways (36). In summary, this thorough review highlights the crucial roles played by metabolites derived from microorganisms in the regulation of anti-tumor immunity, responses to chemotherapy, and outcomes of immunotherapeutic interventions. Through a comprehensive analysis of the complex interrelationships among these metabolites and diverse cancer treatments, this article makes a valuable contribution to the field of cancer biology by laying the groundwork for the development of novel and more efficacious therapeutic strategies in the continuous fight against cancer.

**Table 1:** The role and mechanisms of gut microbiota-derived metabolites in anti-tumor immunity, cancer immunotherapy and chemotherapy.

Gut microbiota-derived metabolites	Metabolite producing bacteria	Actions	Mechanism	Conclusion	Ref.
Indole-3-lactic acid	*Lactobacillus plantarum*	Promote antitumor immunity	Epigenetic regulation of CD8+ T cell immunity	The study demonstrates that indole-3-lactic acid, produced from *Lactobacillus plantarum*, improves colorectal carcinogenesis by epigenetically regulating the immune response of CD8+ T cells.	(24)
Tryptophan	*Lactobacillus* spp.	Suppress anti-tumor immunity	Activation of the AHR	The AHR is activated in TAMs by microbial metabolites generated from tryptophan, leading to the suppression of anti-tumor immunity.	(223)
Butyrate	–	Promote antitumor therapeutic efficacy	Through the ID2-dependent regulation of CD8+ T cell immunity	Butyrate enhances the effectiveness of anticancer treatment by regulating the immune response of cytotoxic CD8+ T cells.	(23)
kynurenine and indole-3-aldehyde	–	Suppress anti-tumor immunity	Activation of the AHR	kynurenine and indole-3-aldehyde produced by the gut microbiota may act as mediators of immunosuppression in premalignant conditions, raising the risk of cancer and potentially as targets for anti-cancer strategies.	(326)
Pentanoate and butyrate	*Megasphaera massiliensis, Megasphaera elsdenii*, and *Faecalibacterium prausnitzii*	Promote antitumor immunity	Modulation of the CD8+ T cell responses	Butyrate and pentanoate influence CD8+ T cell responses, which enhances cancer adoptive immunotherapy.	(22)
Lactate	–	Promote antitumor immunity	Increases stemness of CD8 + T cells	Lactate improves the stemness of CD8+ T cells, which boosts anti-tumor immunity.	(327)
d-lactate	–	Suppress anti-tumor immunity	Modulation of the M2 tumor-associated macrophages	D-lactate alters the immunosuppressive TME for HCC via modifying M2 tumor-associated macrophages.	(328)
Indole-3-lactic acid	*Lactobacillus gallinarum*	Promote antitumor immunity	Promotes apoptosis of cancer cells	*Lactobacillus gallinarum* defends against the development of intestinal tumors by producing indole-3-lactic acid, which can stimulate apoptosis in CRC cells.	(237)
Bile acid	*Clostridium species*	Promote antitumor immunity	Increase of hepatic CXCR6+ natural killer T (NKT)	Liver cancer is regulated by bile acid metabolism controlled by the gut microbiota via NKT cells.	(20)
SCFAs	*B. caecimuris, B. xylanisolvens, and C. bolteae*	Suppress anti-tumor immunity	Increasing Tregs number and impairing the function of CD8+ T cells	SCFAs induce an immunosuppressive state in T cells, which is defined by an increase in regulatory T cells and a decrease in CD8+ T cells.	(21)
Trimethylamine N-oxide (TMAO)	*bacillus* and *paenibacillus ssp.*	Promote antitumor immunity	Potentiated the type I interferon (IFN) pathway	Targeting TMAO may be a possible treatment strategy since TMAO acts as a driver of antitumor immunity.	(269)
TMAO	*Blautia, Ruminococcus, Faecalibacterium, Dorea, Tyzzerella*, and *Roseburia*	Promote antitumor immunity	Activation of the endoplasmic reticulum stress kinase PERK	TMAO promoted pyroptosis in cancer cells by activating the PERK kinase in the endoplasmic reticulum, leading to an increased immune response mediated by CD8+ T lymphocytes against tumors in triple-negative breast cancer *in vivo*.	(260)
Formate	*Fusobacterium nucleatum*	Suppress anti-tumor immunity	Induction of AhR signalling	Microbiome-derived formate increases cancer stemness while inducing AhR signaling, which accelerates CRC tumor expansion.	(241)
Desaminotyrosine (DAT)	*Flavonifractor plautii*	Facilitates immune checkpoint inhibitor treatment	DAT potently enhanced expansion of antigen-specific T cells following vaccination with an IFN-I-inducing adjuvant	Mice given oral supplementation of the microbial metabolite DAT show enhanced anti-CTLA-4 or anti-PD-1 ICI immunotherapy and delayed tumor development.	(29)
Succinic acid	*Fusobacterium nucleatum*	Tumor resistance to immunotherapy	suppressed the cGAS-interferon-β pathway, consequently dampening the antitumor response by limiting CD8^+^ T cell trafficking to the TME	In CRC, *F. nucleatum* and succinic acid cause tumor resistance to immunotherapy, providing information on the interactions between the microbiota, metabolites, and immune system.	(30)
Butyrate	*Faecalibacterium* and *Faecalibacterium*	Limit antitumor effect of CTLA-4 blockade	butyrate restrains anti-CTLA-4-induced up-regulation of CD80/CD86 on dendritic cells and ICOS on T cells, accumulation of tumor-specific T cells and memory T cells.	Elevated concentrations of butyrate and propionate in the bloodstream have been associated with reduced responsiveness to CTLA-4 inhibition and increased regulatory T cells.	(79)
Butyrate	*Akkermansia muciniphila, Bifidobacterium pseudolongum, Bacteroides acidifaciens, Lactobacillus murinus, Faecalibacterium prausnitzii*, and *Faecalibaculum rodentium*	Promotes anti-PD-1 antitumor efficacy	Increased histone 3 lysine 27 acetylation (H3K27ac)	Butyrate, a microbial metabolite, increases anti-PD-1 antitumor effectiveness via altering TCR signaling of cytotoxic CD8 T cells.	(31)
Butyrate	*Roseburia intestinalis*	Boosts anti-PD-1 efficacy	Butyrate directly bound to toll-like receptor 5 (TLR5) receptor on CD8+ T cells to induce its activity through activating nuclear factor kappa B (NF-κB) signalling.	The production of butyrate by *Roseburia intestinalis* enhances the effectiveness of anti-PD-1 treatment in colorectal cancer by stimulating cytotoxic CD8+ T cells.	(28)
Butyric acids, α-ketoglutaric acid, N-acetyl-l-glutamic acid and pyridoxine	Lacticaseibacillus rhamnosus	Enhanced the antitumor response to anti-PD-1 therapy	Promotion of the infiltration and activation of cytotoxic T lymphocytes (CTLs) and suppressing the function of Tregs in the TME.	The probiotic strain *Lacticaseibacillus rhamnosus* Probio-M9 improved the effectiveness of anti-PD-1 treatment in fighting tumors by altering the composition of metabolites in the intestines.	(329)
Inosine	*Bifidobacterium pseudolongum*	Modulates response to checkpoint inhibitor immunotherapy	T cell expression of the adenosine A2A receptor	Immunotherapy-induced impaired gut barrier function led to an increase in inosine translocation systemic translocation and the activation of antitumor T cells. The impact of inosine relied on the presence of the adenosine A2A receptor in T cells and necessitated co-stimulation.	(330)
Tryptophan	*Lactobacillus reuteri*	Facilitates immune checkpoint inhibitor treatment	Released aryl hydrocarbon receptor (AhR) agonist indole-3-aldehyde (I3A) and CD8 T cells within the tumor microenvironment.	The treatment with immune checkpoint inhibitors is facilitated by the dietary tryptophan metabolite produced by intratumoral *Lactobacillus reuteri*.	(331)
Trimethylamine N-oxide (TMAO)	*Bacillus and Paenibacillus genus*	Boosts responses to immune checkpoint inhibitor treatment	Potentiated type-I interferon (IFN) pathway	In pancreatic cancer, the microbiome-derived metabolite TMAO promotes immunological activation and increases responses to immune checkpoint blocking.	(269)
Indole-3-carboxylic acid (ICA)	*Lactobacillus gallinarum*	Improved anti-PD1 efficacy	Inhibiting regulatory T cells through modulating IDO1/Kyn/AHR axis	IDO1/Kyn/AHR axis modulation by *Lactobacillus gallinarum*-derived ICA enhanced anti-PD1 effectiveness in CRC by inhibiting CD4+Treg differentiation and promoting CD8+T cell activity.	(212)
Indole-3-lactic acid (ILA)	Lactobacillus reuteri	Chemopreventive effects	Targeting RAR-related orphan receptor γt (RORγt).	ILA exhibited anti-tumorigenic properties via the downregulation of the IL-17 signaling pathway. This implies that CRC chemoprevention techniques might be complemented by therapies using *Lactobacillus reuteri* or ILA.	(37)
ILA	*Bacteroides fragilis* and *Bacteroides thetaiotaomicron*	Promotes the chemotherapy effects	Via downregulation of the reactive oxygen species (ROS)-degrading enzymes glutathione peroxidase 3 and glutathione peroxidase.	In pancreatic cancer, 3-IAA produced from microbiota affects the effectiveness of treatment.	(35)
Lactate	*Lactobacillus iners*	Chemoradiation resistance	Increased L-lactate production	Lactic acid bacteria in the TME may affect tumor metabolism and lactate signaling pathways, resulting in treatment resistance.	(36)

## 2 Gut microbiota and their metabolites as a key player in health and diseases

The gut microbiota encompasses a diverse and extensive array of microorganisms residing within the human gastrointestinal tract (38). This intricate network comprises bacteria, viruses, fungi, and archaea, collectively exerting a profound influence on health and contributing to the onset and progression of various diseases (39). The metabolites generated by these microbial communities play a pivotal role in fostering a mutually beneficial relationship between the host and microbes, significantly influencing the overall health of the human body (40–42).

Metabolites, essential biomolecules involved in energy generation and transformation, pose significant challenges to metabolic research due to their reactivity, structural variability, and wide concentration spectrum (43). In the realm of biology, primary metabolites contribute to fundamental life cycle functions, such as growth, differentiation, and reproduction, while secondary metabolites serve various purposes, including signaling, protection, and activities unrelated to the life cycle (43). The solubility of compounds, distinguishing between water-soluble and insoluble, plays a crucial role in applied metabolomics (43). Moreover, the gut microbiota plays a pivotal role in metabolizing ingested food or host-produced substances into molecules that either nourish the gut microorganisms or pose harm to the host’s cells. As a result, the presence of metabolites is intricately linked to the metabolic functions of bacteria (44, 45).

The gut microbiota plays a dual role in producing compounds that can either benefit or harm the host. Microbiota in the colon produce a diverse array of metabolites, including tryptophan catabolites, SCFAs, and polyamines (46, 47). In recent research, the substantial influence of metabolites derived from microbes on both health and disease has been emphasized. Butyrate, a microbial product, has been thoroughly researched and shown to be extremely advantageous. It is produced by four well-known processes, with the acetyl-CoA pathway being the primary one, and the lysine, glutarate, and succinate pathways playing a less significant part (48). Butyrate offers a range of therapeutic advantages, serving not only as the primary energy source for luminal colonocytes but also exerting systemic effects. The advantages include preserving the protective coating of mucous membranes throughout the entire body, regulating immunological responses at both local and systemic levels, and preventing abnormal cell proliferation (49, 50). In addition, butyrate promotes the production of mucus by goblet cells, facilitates the creation of immunoglobulins, and increases the secretion of antimicrobial peptides (50, 51). Additionally, it enhances the antibacterial capacities by facilitating the conversion of proinflammatory M1 macrophages into resolution-phase M2 macrophages (52).

TMAO has emerged as a pivotal factor in the development of cardiovascular diseases. Wang et al. were among the first to discover a strong relationship between TMAO levels in the body and the amount of coronary atherosclerosis and cardiac risk (53). Based on this, Tang et al. performed a research that found that elevated blood TMAO levels were substantially related with an increased risk of severe adverse cardiovascular events, a link that remained significant even after controlling for conventional risk variables (54). The negative effects of TMAO on heart failure are diverse, including both direct and indirect effects. These include inducing cardiac hypertrophy and fibrosis, activating inflammatory pathways that lead to endothelial dysfunction, initiating pathological ventricular remodeling, and inducing renal interstitial fibrosis (55, 56). Several therapies have been investigated to modify the interaction between gut microbiota, TMAO, and cardiovascular disease. These therapies vary from dietary changes to the prescription of probiotic supplements targeted at lowering plasma TMAO levels. Furthermore, research is being conducted to find possible enzyme targets for drugs to reduce TMAO formation (57–60).

Indole research demonstrates its powerful antimicrobial capabilities. It has been shown to be effective against a wide range of bacteria, including *Staphylococcus aureus*, *Salmonella*, *Lactobacillus*, *Escherichia coli*, and *Bacillus cereus*. Furthermore, indole-ethanol has been found as a bacteriophage replication inhibitor in select bacterial strains and has been demonstrated to limit the growth of parasitic protozoa (61, 62). Tryptophan metabolites, including indole, exert influence on both the innate and adaptive immune systems by binding to the aryl hydrocarbon receptor (AHR) present on immune cells such as DCs and T-cells (63). The systemic impact of tryptophan and its metabolites extends to hormone production, showcasing anti-inflammatory characteristics. Indole enhances glucagon-like peptide 1 release by acting as a signaling molecule in the colon’s L cells. This, in turn, influences insulin release from pancreatic B-cells, decreases appetite, and delays stomach emptying (64, 65).

Putrescine, spermidine, and spermine stand out as crucial metabolites synthesized by the gut microbiota, exerting a profound influence on the overall well-being of the host. Research has elucidated that polyamine (PAs), such as putrescine, spermidine, and spermine, possess antioxidant properties and can impede the generation of inflammatory cytokines. Notably, PAs have the capacity to impact the integrity of the intestinal mucosal barrier. Probiotic interventions have demonstrated the potential to enhance the host’s lifespan by mitigating chronic low-grade inflammation induced by heightened levels of physical activity. These effects encompass increased resistance to oxidative stress (66). In recent epidemiological studies, substantial evidence has emerged establishing a correlation between increased polyamine intake, specifically spermidine, and a decreased risk of cardiovascular events and mortality (66, 67). Furthermore, recent studies have revealed that reduced amounts of spermidine and spermine might potentially result in an accumulation of dcAdoMet, which in turn leads to a decline in DNA methylation levels (68).

In conclusion, the role of microbial-derived metabolites in health and disease is complex and multifaceted. Maintaining a balanced and diverse gut microbiota that produces beneficial metabolites is critical to overall health, while disruption of this system can contribute to the development of various diseases. Further research in this field holds great promise for understanding the intricate interplay between the microbiome and human health and may lead to innovative approaches for preventing and treating a range of health conditions.

Next, we will examine the role of microbial metabolites in anti-tumor immune responses, tumor immunotherapy and the effect of these metabolites on the response to chemotherapy. To this aim, we will first provide a brief description of each metabolite, and then we will discuss the latest findings regarding the role of these microbiota-derived metabolites in anti-tumor immune responses, tumor immunotherapy, and the response to chemotherapy. In **Table 1**, the mechanistic roles of microbe-derived metabolites in anti-tumor immune responses, cancer immunotherapy, and chemotherapy are presented, which will be thoroughly examined in the subsequent sections.

## 3 Microbiota-derived SCFAs

Microbiota-derived SCFAs are organic acids consisting of carbon atoms ranging from two to five. Their formation arises from the anaerobic fermentation of indigestible polysaccharides, such as dietary fiber and resistant starch, by bacteria residing in the human gastrointestinal tract (69, 70). SCFAs are negatively charged molecules consisting of carbon chains that vary in length from 1 to 6. The most abundant SCFAs include acetate (C2), propionate (C3), and butyrate (C4). Furthermore, SCFAs can be generated by the metabolic breakdown of amino acids (71). In addition, SCFAs can be generated by the metabolic breakdown of amino acids, with around 1% of the bacterial population in the large intestine employing these metabolic pathways (9, 72, 73). SCFAs are mostly absorbed by colonocytes after production, which is aided by H+-linked monocarboxylate transporters (MCTs) and sodium-linked monocarboxylate transporters (SMCTs) (74). SCFAs play a pivotal role in shaping the composition of the intestinal microbiota (75), enhancing the function of the intestinal epithelial barrier (76), and exhibiting positive effects in slowing the progression of various diseases, including chronic kidney disease (77), inflammatory bowel disease (IBD) (78), cancer (79–81), obesity (82). SCFAs, defined by their chain length of 1 to 6 carbon atoms, are classified as saturated fatty acids (83). They exert their influence on various cell types, playing a crucial role in regulating essential biological processes such as host metabolism, intestinal function, and immunology (84–86). The molar ratio of acetate, propionate, and butyrate in the human colon and feces is approximately 60:20:20 (87, 88). SCFAs are generated through the bacterial fermentation process. Thus, the primary reason for the varying ratios of acetate, propionate, and butyrate lies in the breakdown of distinct bacterial species (89).

The activity of SCFAs is mediated through two signal transduction mechanisms: the inhibition of histone deacetylase (HDAC) and the activation of G protein-coupled receptors (GPCRs) (90). Given that HDAC-mediated epigenetic modifications are crucial for gene expression (91), the suppression of HDAC by SCFAs influences the progression of metabolic, cancerous, and immune-related disorders (92–94). GPCRs, specifically GPR43 (also known as free fatty acid receptor 2, FFAR2), GPR41 (FFAR3), and GPR109A (also known as hydroxycarboxylic acid receptor 2, HCA2), have been identified as receptors for SCFAs. These GPCRs play significant roles in regulating metabolism and inflammation (87, 95, 96). For instance, the activation of HCA2 by butyrate is crucial for maintaining intestinal homeostasis (97). HCA2 is mainly expressed in intestinal epithelial cells (IECs), adipose tissue, and activated macrophages within adipose tissue. It interacts with butyrate but does not get activated by either propionate or acetate (98). Interestingly, the levels of HCA2 mRNA and protein in intestinal epithelial cells (IECs) are significantly lower in germ-free mice compared to conventional mice, owing to the lack of gut microbiota. This reduction is reversed when the intestinal tract of germ-free mice is re-colonized with bacteria (99). In IECs, the binding of a ligand to HCA2 activates the NOD-, LRR- and pyrin domain-containing protein 3 inflammasome, which facilitates the maturation and secretion of IL-18 (100, 101). HCA2 inhibits both basal and LPS-induced nuclear factor-kappa B (NF-κB) activation in normal and cancerous colonocytes (97). In DCs, HCA2 activation reduces IL-6 levels, boosts IL-10 levels, and increases the expression of RALDH1, an enzyme responsible for converting retinol to retinoic acid (RA). RA is essential for the proliferation and function of regulatory T cells (Tregs), particularly in the gut, in both mice and humans (102–104). Additionally, in neutrophils, activation of HCA2 by niacin elevates levels of the pro-apoptotic protein Bcl-2 associated agonist of cell death (105). Overall, these findings highlight HCA2’s significant role in nutrient sensing and protecting the host from pro-inflammatory challenges across various cell types through different signaling pathways.

Innate lymphoid cells (ILCs) are a diverse group of cells primarily found in non-lymphoid peripheral tissues. This family includes five distinct subtypes: natural killer (NK) cells, ILC1s, ILC2s, ILC3s, and lymphoid tissue inducer (LTi) cells. These subpopulations share similarities with T cell subsets in terms of their cytokine production and transcription factor expression profiles (106, 107). ILCs are recognized for their ability to change functions and phenotypes in response to varying environmental signals, a phenomenon referred to as functional plasticity (108, 109). Their tissue-resident characteristics enable ILCs to quickly react to specific stimuli within tissues and coordinate both innate and adaptive immune responses during infections (110, 111). Beyond their traditional role in enhancing inflammatory responses, there is growing acknowledgment of the immunoregulatory functions of ILCs in a range of diseases, including cancer (112, 113). The relationship between ILCs and the microbiota is intricate. Microbiota influences ILCs indirectly through signals from accessory cells, including DCs and IECs, as well as via the signaling pathways associated with microbiota-derived metabolites and related dietary components. Conversely, ILCs also play a role in regulating the microbiota by secreting effector cytokines such as IFN-γ from NK cells, IL-4 from ILC2s, and IL-22 from ILC3s, in addition to the involvement of T and B cells (114). The relationship between ILCs and microbial metabolites, especially SCFAs, is a burgeoning field in immunology with important implications for gut health and disease (115–117). For instance, Kim et al. investigated how microbial metabolites shape the landscape of intestinal immune cells, with a particular focus on ILC3s in Peyer’s patches (PPs) (116). heir findings revealed that specific pathogen-free (SPF) mice have fewer NKp46+ ILC3s in their terminal ileal PPs compared to those in jejunal PPs, a difference that was not observed in antibiotic-treated mice (116). This suggests that specific components of the microbiota may affect the distribution of ILC3s. Additionally, higher concentrations of butyrate, a microbial metabolite, were detected in the terminal ileal PPs of SPF mice compared to both jejunal PPs of SPF mice and terminal ileal PPs of antibiotic-treated mice (116). The study demonstrated that butyrate suppresses NKp46+ ILC3s in terminal ileal PPs, leading to reduced Csf2 expression, fewer Tregs, and increased proliferation of antigen-specific T cells (116). This indicates that butyrate derived from the microbiota negatively regulates NKp46+ ILC3s, thereby modulating the immune environment and enhancing specific immune responses in the terminal ileal PPs. Overall, this research highlights the crucial role of microbiota-derived metabolites in regional immune regulation in the gut. In the following section, we will delve into the functions of butyrate, propionate, and acetate in anti-tumor immunity, cancer immunotherapy, and chemotherapy.

### 3.1 Butyrate

Numerous commensal bacteria, such as *Clostridium cluster* IV and XIVa, as well as *Faecalibacterium prausnitzii*, play a crucial role in promoting the production of butyrate, subsequently absorbed by the intestinal epithelial cells (IECs) of the human host (84, 118, 119). The synthesis of butyrate from carbohydrates occurs through glycolysis, involving the fusion of two Acetyl-CoA molecules to form acetoacetyl-CoA, which is then gradually reduced to yield butyryl-CoA. The final stage in the synthesis of butyrate from butyryl-CoA can be achieved through two distinct methods: either via the butyryl-CoA: acetate CoA-transferase pathway or through the phospho-butyrate and butyrate kinase pathways (72). Butyrate plays a pivotal role in maintaining the integrity of the colonic mucosa in BALB/c mice, thereby resisting colitis and preventing the onset and progression of cancer. Its mechanism involves the regulation of cell proliferation, apoptosis, and differentiation (85, 120, 121). Furthermore, butyrate enhances the function of the intestinal barrier and reduces inflammation in the intestines of mice with intestinal disorders. This is accomplished through interactions with GPCRs and the suppression of HDACs (122). The data strongly suggest that butyrate contributes to the enhancement of the intestinal barrier’s integrity, resulting in improvements in colonic inflammation and the inhibition of colon cancer incidence and progression. This section has delved into the multifaceted role of butyrate within the context of anti-tumor immunity, cancer immunotherapy, and chemotherapy.

#### 3.1.1 The role of butyrate in anti-tumor immunity

He et al. studied the effect of gut microbial metabolites, especially butyrate, on the efficacy of oxaliplatin in cancer treatment via the control of CD8+ T cell activity in their study (23). Their research encompasses both *in vitro* and *in vivo* tests, shedding light on the molecular processes underlying the observed effects. Moreover, the research extends its findings to real cancer patients, establishing clinical significance for the observed phenomena. The gut microbiome has the potential to influence the host’s immune system, and manipulating the gut microbiota could potentially enhance the body’s ability to combat tumors. Chemotherapy may induce dysbiosis, leading to heightened Th1 and Th17 immune responses, which, in turn, can impact the effectiveness of chemotherapy (123). CD8+ T cells play a pivotal role in directly eliminating tumor cells and are considered a primary component in the immune response against tumors. The activity of CD8+ T cells may also be influenced by myeloid cells and CD4+ T cells. However, it remained unclear whether the microbiota might directly modulate the activity of antitumor cytotoxic T cells or indirectly influence the CD8+ T cell response through myeloid or Th1 and Th17 cells. The research conducted by He et al. illustrates that the gut microbiota may directly enhance the immune response of antitumor CD8+ T cells and improve the effectiveness of chemotherapy by generating specific metabolites, notably butyrate (23). Further research is imperative to ascertain whether the gut microbiota influence the response of antitumor CD8+ T cells through interactions with other immune cells or other chemicals within the gut microbiome (23). Numerous studies have consistently demonstrated a robust correlation between the presence of fecal butyrate and the incidence of CRC (124–126). Additionally, He et al. observed a potential favorable correlation between blood butyrate levels and the response to chemotherapy in patients (23). Recent validation has been provided for the connection between SCFAs and the therapeutic effects of PD-1 inhibition in cancer patients (127). A study conducted by He et al. emphasizes the significant influence of gut microbial metabolites, specifically butyrate, in improving the effectiveness of oxaliplatin in cancer treatment (23). The direct enhancement of the immune response of cytotoxic CD8+ T cells against tumors, both in laboratory settings and living organisms, was evident upon the administration of butyrate (23). Previous research has indicated that animals lacking inhibitor of DNA binding protein 2 (ID2) in CD8+ T cells are more susceptible to bacterial infections due to compromised development of effector and memory CD8+ T cells (128–131). According to He et al., the augmentation of CD8+ T cell responses induced by butyrate operates through a mechanism reliant on ID2 (23). Furthermore, it was demonstrated that the administration of butyrate led to the promotion of the IL-12 signaling pathway, subsequently influencing the regulation of CD8+ T cell activity (23). He et al. provides detailed insights into how butyrate enhances the immune response of antitumor CD8+ T cells, primarily through mechanisms involving ID2 and the IL-12 signaling pathway. However, these findings could benefit from further elaboration on the interactions with other immune cells and signaling molecules. The observation of a favorable correlation between blood butyrate levels and chemotherapy response in patients underscores the potential for clinical applications. Yet, translating these findings into standardized treatment protocols will require extensive clinical trials and validation. The study suggests that chemotherapy-induced dysbiosis can lead to heightened Th1 and Th17 responses, impacting treatment effectiveness. This highlights the importance of considering the gut microbiome’s health in cancer therapy planning. However, the specific pathways through which dysbiosis influences these immune responses warrant further investigation.

Yang et al. conducted an investigation into the role of gut microbiota in the anticancer effects of ionizing radiation (IR) and identified a novel mechanism involving the butyrate-mediated reduction of local type I interferon (IFN) production (132). The research establishes a correlation between the composition of gut microbiota, levels of butyrate, and the effectiveness of IR in mitigating tumor development. To manipulate butyrate-producing gut bacteria, the study utilized antibiotic therapy and oral administration of specific microorganisms, revealing a potential treatment avenue for enhancing tumor radiation sensitivity. The administration of vancomycin, an antibiotic targeting Gram-positive bacteria, resulted in a reduction of butyrate-producing gut bacteria (132). This decrease in butyrate-producing bacteria was associated with an increase in antitumor responses to IR (132). Additionally, the researchers found that oral treatment with *Lachnospiraceae*, a vancomycin-sensitive bacterial family, led to elevated systemic and intratumoral butyrate levels (132). This heightened butyric acid was correlated with reduced IR efficiency in germ-free mice (132). Prior studies have indicated that the IR-induced cytotoxic T cell response is significantly reliant on tumor-associated myeloid cells and stimulator of interferon genes (STING) activation (133–135). IR has the potential to stimulate an enhanced tumor-specific cytotoxic T cell response dependent on DCs (136). In a previous report, Deng et al. highlighted that IR triggers STING activation in intratumoral DCs, resulting in increased IFN-I expression and an enhanced cytotoxic T cell response against tumor antigens (136). Building on this, Yang et al. discovered that local administration of butyrate inhibits STING-activated type I interferon expression in DCs (132). The activation of STING recruits’ serine-threonine kinase (TBK1) to phosphorylate the IFN regulatory factor 3 (IRF3), ultimately leading to IFN-I production (79, 137, 138). Their results unveiled that this inhibition occurs through the blockade of TBK1 and IRF3 phosphorylation (132). Consequently, the suppression of IFN-I expression diminishes IR-induced tumor-specific cytotoxic T cell immune responses (132). These findings demonstrate that sodium butyrate (NaBu) hinders downstream STING activation by interfering with the phosphorylation of TBK1 and IRF3 (132). Importantly, the findings of Yang et al. emphasize that the impact of butyrate on immune responses is not a result of directly shielding tumor cells from radiation. Instead, its impact lies in modulating the immune microenvironment, specifically through the inhibition of IFN-I expression. Yang et al. expand on previous findings by exploring the impact of butyrate on the STING pathway and IFN-I expression. Their discovery that butyrate inhibits STING-activated type I interferon expression in DCs by blocking TBK1 and IRF3 phosphorylation is crucial. However, the downstream effects of this inhibition and its broader implications on the immune microenvironment require further elucidation. To translate these findings into clinical practice, studies involving human subjects are essential. This will help validate whether the observed mechanisms in mice apply to human physiology. Also, expanding the analysis to include a wider range of gut microbiota and metabolites could uncover additional factors influencing IR effectiveness. All in all, conducting longitudinal studies to observe the long-term effects of manipulating butyrate-producing bacteria on cancer treatment outcomes would provide deeper insights.

All in all, He et al. investigated how gut microbial metabolites, especially butyrate, enhance the efficacy of oxaliplatin in cancer treatment by regulating CD8+ T cell activity, demonstrating significant effects in both laboratory and clinical settings. Their research highlights that butyrate improves the immune response of CD8+ T cells through mechanisms involving ID2 and the IL-12 signaling pathway. They also observed a positive correlation between blood butyrate levels and chemotherapy response, suggesting potential clinical applications. However, chemotherapy-induced dysbiosis can lead to heightened Th1 and Th17 responses, affecting treatment outcomes. Yang et al. further explored butyrate’s role, finding it inhibits STING-activated type I interferon expression in DCs, thus modulating the immune microenvironment and enhancing radiation therapy effectiveness. These findings underscore the importance of gut microbiota in cancer therapy, necessitating further research to validate these mechanisms in humans and explore additional microbial influences on treatment efficacy.

#### 3.1.2 The role of butyrate in cancer immunotherapy

Coutzac et al. conducted a study investigating the impact of systemic SCFAs, particularly butyrate and propionate, on the therapeutic outcomes of Cytotoxic T-lymphocyte associated protein 4 (CTLA-4) immune checkpoint inhibitor treatment in both murine models and individuals with advanced cancer (79). The primary objective of the study is to unveil the molecular mechanisms through which gut microbiota-derived metabolites, specifically SCFAs, influence immune responses and the effectiveness of anti-CTLA-4 treatment (79). The data indicate a correlation between elevated blood levels of butyrate and propionate, resistance to CTLA-4 inhibition, and alterations in Treg cell proportions. SCFA, especially butyrate, consistently exhibits immunomodulatory properties (as does propionate), primarily through the induction of Treg cells (139). CTLA-4 inhibition leads to an upregulation in the expression of co-stimulatory molecules CD80 and CD86 in mice, a process hindered by the oral administration of sodium butyrate. The research delves into how the composition of the gut microbiota influences the therapeutic efficacy of immune checkpoint inhibitors, specifically CTLA-4 inhibition, in individuals with advanced cancer (79). Coutzac et al. demonstrated a correlation between blood levels of butyrate and propionate and resistance to CTLA-4 blockade (79). Elevated SCFA levels are also associated with an increased number of Treg cells, suggesting a potential immunosuppressive effect (79). In animal models, butyrate hinders the CTLA-4-induced upregulation of CD80/CD86 on DCs and ICOS on T cells (79). Additionally, butyrate inhibits the formation of tumor-specific T cells and memory T cells, potentially hindering the immune response against cancer (79). Elevated levels of butyrate are associated with reduced IL-2 impregnation, indicating a potential regulatory role in immune responses (79). This study suggests that systemic short-chain fatty acids, especially butyrate and propionate, may act as regulators, diminishing the anticancer effects of CTLA-4 inhibition in cancer patients. The correlation between higher SCFA levels and medication resistance, along with changes in Treg cell proportions, highlights a complex interplay between gut microbiota-derived chemicals and immunological checkpoint suppression. The findings offer insights into the molecular processes through which SCFAs modulate immune responses, emphasizing their potential as regulators of therapeutic outcomes in cancer patients undergoing CTLA-4 inhibition. Further exploration in this realm could contribute to devising strategies for optimizing immune checkpoint inhibitor therapy.

Zhu et al. investigated the impact of the microbial metabolite butyrate on modifying antitumor immunity, specifically focusing on PD-1 expression on CD8+ and V9 V2 (V2+) T cells in patients with non-small cell lung cancer (NSCLC) (31). One possible explanation for this observation is that signals derived from commensal *Bifidobacterium* modulate the activation of DCs, consequently supporting enhanced effector function in tumor-specific CD8+ T cells (140). Conversely, non-responders exhibit a lower abundance of SCFA-producing bacteria, such as *Faecalibacterium* and *Akkermansia muciniphila*, compared to responders to anti-PD-1 immunotherapy (141, 142). Zhu et al. demonstrated a positive correlation between serum butyric acid levels and PD-1 expression on circulating CD8+ and V2+ T lymphocytes in NSCLC patients (31). Furthermore, responder NSCLC patients, those showing a favorable response to therapy, exhibited higher levels of blood acetic acid, propionic acid, and butyric acid than non-responders (31). Their findings indicated that depleting gut microbiota from tumor-bearing animals led to reduced butyrate levels in both feces and blood (31). This suggests a connection between gut microbiota and systemic butyrate levels.

Previous studies have demonstrated that SCFAs, especially butyrate, stimulate the generation of regulatory T cells (Treg cells) outside the thymus, contribute to the development of Treg cells in the colon, and are regulated by HDAC (143–145). Zhu et al. made the discovery that butyrate enhances the activation of CD28 and PD-1 in cytotoxic CD8+ and Vδ2+ T lymphocytes by promoting histone 3 lysine 27 acetylation at the promoters of Cd28 and Pdcd1 genes (31). This mechanism facilitates the upregulation of PD-1/CD28, thereby enhancing the effectiveness of anti-PD-1 treatment. T-cell activation requires two signals: an initial signal triggered by the antigen from the T cell receptor (TCR) and a subsequent signal from co-stimulatory receptors (146). Previous studies have indicated that the TCR/CD28/Ca2+ signaling pathway has the capacity to amplify the antigen sensitivity of T lymphocytes (147, 148). According to the current model of TCR activation, upon TCR binding, Lck is activated, leading to the phosphorylation of the CD3 coreceptor complex and ζ-chains of the TCR. This activation also initiates the activation of the ζ-chain-associated protein Zap70. Subsequently, the activated tyrosine kinase Zap70 phosphorylates the membrane adaptor Lat, which then recruits various Src homology-containing proteins, including phospholipase C-γ1 (PLC-γ1) (148–150). Therefore, PLC-γ1 plays a crucial and essential role in the conventional TCR signaling pathway (31). Zhu et al. found that butyrate facilitates the phosphorylation of PLC-γ1 and enhances the synthesis of IFN-γ and tumor necrosis factor α (TNFα) in CD8+ T cells during TCR activation (31). Importantly, the co-administration of anti-PD-1 and butyrate resulted in more potent immune responses against tumors compared to using either anti-PD-1 or butyrate alone in mouse models with melanoma. This study provides crucial insights into the influence of the gut microbiota metabolite, butyrate, on the efficacy of anti-PD-1 immunotherapy in NSCLC patients. The positive correlation between serum butyric acid levels and PD-1 expression, as well as the association with treatment response, suggests a potential role for butyrate as a therapeutic biomarker. The mechanistic exploration reveals that butyrate enhances the expression of PD-1/CD28 and promotes antitumor cytokine expression by modulating the TCR signaling pathway in cytotoxic CD8+ T cells. Overall, these findings position butyrate as a promising candidate for enhancing antitumor immunity and optimizing the effectiveness of anti-PD-1 immunotherapy in cancer patients.

Kang et al. investigated the involvement of *Roseburia intestinalis*, a probiotic species renowned for its anti-inflammatory characteristics, in the development of CRC and the efficacy of immunotherapy (28). The primary goal was to assess the prevalence of Roseburia intestinalis in individuals with CRC, scrutinize its impact in animal models of CRC, and unveil the underlying mechanisms through which *Roseburia intestinalis* and its metabolite, butyrate, influence tumor development and response to anti-PD-1 immunotherapy (28). Previous studies have indicated a reduction in the presence of *Roseburia intestinalis* in the gut microbiota of individuals with CRC (151–153). There is also evidence suggesting a negative correlation between butyrate and the incidence of CRC (154). Moreover, an increasing body of information highlights the various protective mechanisms of butyrate against the development of CRC. Butyrate can diminish tumor cell proliferation by acting as a histone deacetylase inhibitor (155). *Clostridium butyricum*, the main producer of butyrate in the human gut, reduces the occurrence of CRC by downregulating the oncogenic WNT signaling pathway (156). In their recent investigation, Kang et al. observed a substantial reduction of *Roseburia intestinalis* in the fecal samples of patients with CRC compared to healthy individuals (28). The administration of *Roseburia intestinalis* suppressed the development of tumors in ApcMin/+ mice and animals with azoxymethane (AOM)-induced CRC (28). Kang et al. found that *Roseburia intestinalis* has the capacity to enhance gut barrier function, increase intestinal permeability, and elevate the expression of tight junction proteins (28). Furthermore, it has been demonstrated that *Roseburia intestinalis* produces butyrate as its functional metabolite (28). Consequently, the researchers investigated the correlation between *Roseburia intestinalis* or butyrate and the efficacy of anti-PD-1 treatment in two orthotopic mouse models featuring tumors with distinct CRC subtypes, namely microsatellite instability (MSI)-high or microsatellite instability (MSS) (28). In their study, Kang et al. observed that *Roseburia intestinalis* or butyrate stimulates the development of cytotoxic CD8+ T cells expressing granzyme B, IFN-γ, and TNF-α in orthotopic animal models of CRC (28). Additionally, the presence of *Roseburia intestinalis* or butyrate significantly enhances the effectiveness of anti-PD-1 immunotherapy in mice with CRC exhibiting microsatellite instability (MSI)-low (28). Furthermore, the researchers delved into the mechanism through which butyrate produced from *Roseburia intestinalis* interacts with CD8+ T cells possessing anticancer properties (28). A prior investigation suggested that inhibiting GPR did not impede the impact of butyrate on the functionality of CD8+ T lymphocytes (23). Toll-like receptor 5 (TLR5), a receptor found on epithelial and immune cells that selectively detects flagellin on bacteria (157), lacks substantial research on its interaction with microbial metabolites. Kang et al. revealed that butyrate directly binds to the TLR5 receptor on CD8+ T cells (28). This binding activates TLR5 through the nuclear factor kappa B (NF-κB) signaling pathway, thereby enhancing the functional activity of CD8+ T lymphocytes.

The findings of this research illustrate that *Roseburia intestinalis* protects against the development of CRC by generating butyrate, which subsequently boosts the efficacy of anti-PD-1 immunotherapy. The depletion of *Roseburia intestinalis* in CRC patients suggests a potential role for this probiotic species in CRC prevention. The mechanisms involve the induction of functional cytotoxic CD8+ T cells and the direct interaction of butyrate with TLR5 on these T cells. The findings suggest that *Roseburia intestinalis* or butyrate could serve as potential adjuvants to augment the efficacy of anti-PD-1 immunotherapy in CRC. In summary, the importance of SCFAs, particularly butyrate, lies in their diverse beneficial effects on gut health, immune modulation, and potential therapeutic applications in various diseases, including cancer. The studies discussed provide insights into the intricate relationship between gut microbiota-derived metabolites and the host immune system, paving the way for novel therapeutic strategies in cancer treatment.

In conclusion, the research conducted by Coutzac et al., Zhu et al., and Kang et al. underscores the critical role of SCFAs, particularly butyrate, in shaping immune responses and therapeutic outcomes in cancer treatment. Coutzac et al. revealed that elevated levels of butyrate and propionate correlate with resistance to CTLA-4 inhibition, indicating their immunosuppressive potential. Conversely, Zhu et al. demonstrated that butyrate enhances antitumor immunity by upregulating PD-1 expression on CD8+ T cells, thus improving responses to anti-PD-1 therapy in non-small cell lung cancer. Similarly, Kang et al. highlighted the protective effects of the probiotic Roseburia intestinalis, which produces butyrate and promotes effective cytotoxic T cell responses against CRC. Collectively, these findings emphasize the complex interplay between gut microbiota-derived metabolites and immune modulation, suggesting that SCFAs like butyrate may serve as valuable biomarkers and therapeutic adjuncts to enhance the efficacy of immune checkpoint inhibitors in cancer treatment. Further research is essential to fully elucidate these mechanisms and develop targeted strategies for optimizing immunotherapy in cancer patients.

### 3.2 Acetate

Acetate predominantly originates in the animal colon through the anaerobic breakdown of dietary fibers by bacteria. Various bacteria, including *Akkermansia muciniphila* and *Bacteroides* spp., contribute to the production of acetic acid (85, 87, 158–160). This process involves the conversion of acetyl-CoA, generated by glycolysis, to acetate. Subsequently, enzymatic transformation to butyrate occurs through butyryl-CoA:acetyl-CoA transferase (87). The highest concentration of this fermentation product is found in the proximal colon, where it is absorbed by IECs or transferred to the blood through the intestinal epithelium and rapidly absorbed by the liver via the hepatic portal vein (90, 139). Acetate exerts various effects on tissues and organs (120). It serves as a biofuel and nutritional source for tumor cells and is implicated in lipid formation (161). In both animals and humans, acetate plays a regulatory role in energy balance and metabolic homeostasis. It protects mitochondria from oxidation and stress, influences immunity, and modulates body weight and insulin sensitivity through its effects on glucose and lipid metabolism (162–165). However, further research is needed to delve into the mechanisms by which acetate functions in different disorders and to identify specific metabolic alterations associated with acetate utilization.

Recent revelations highlight the significant role of acetate in cancer etiology (166–168). Tran et al. (166) delves into the potential therapeutic implications of acetate in cancer treatment, focusing on its impact on the expression of the poliovirus receptor (PVR/CD155), an immunological checkpoint ligand in colon cancer cells. Their findings indicate that acetate therapy inhibits PVR/CD155 expression, leading to heightened effector responses of CD8+ T cells (166). This action is achieved by blocking the PI3K/AKT pathway (166). The study further reveals that acetate therapy enhances CD8+ T cell effector responses, suggesting a potential augmentation in anti-tumor immunity (4). The results suggest that maintaining specific acetate concentrations could serve as a complementary approach in cancer therapy, potentially enhancing response rates by modifying immunological checkpoint expression and promoting anti-tumor immunity (166). The data imply that maintaining specific acetate concentrations might be a valuable supplementary strategy for improving anti-tumor immunity within the cancer microenvironment. The proposition that maintaining specific acetate concentrations could supplement current cancer therapy regimens opens up new avenues for research and clinical testing.

In conclusion, acetate emerges as a crucial metabolite with multifaceted roles in health and disease, particularly in cancer dynamics. Generated primarily in the animal colon through bacterial fermentation of dietary fibers, acetate significantly influences metabolic processes, immunity, and energy balance. Recent findings highlight its therapeutic potential, especially in colon cancer, where acetate therapy has been shown to inhibit the expression of the immunological checkpoint ligand PVR/CD155, thereby enhancing the effector responses of CD8+ T cells and potentially boosting anti-tumor immunity. These insights suggest that strategically maintaining acetate concentrations may serve as an innovative adjunct to current cancer therapies, offering a promising approach to improve patient outcomes. Further research is warranted to fully elucidate the underlying mechanisms of acetate’s action in various disorders and to explore its potential as a therapeutic agent in clinical settings.

#### 3.2.1 The role of acetate in anti-tumor immunity

Qiu et al. conducted a study examining the impact of acetate on restoring effector function in glucose-restricted CD8+ T cells, a common occurrence in the TME (168). Adequate nutrient availability is crucial for T cells to activate proper metabolism, facilitating effector activities during infections and malignancies (169–171). According to Qiu et al., acetate plays a role in rescuing effector activity in glucose-restricted CD8+ T cells, addressing their hypo-responsiveness during cancer (168). It is important to mention that local microenvironments and niches may have greater concentrations of acetate in comparison to blood levels. Recent evidence suggests that SCFAs obtained from food, such as acetate, boost the effectiveness of CD8+ T cells via enhancing cellular metabolism (172). Qiu et al. discovered that acetate induces increased histone acetylation and chromatin accessibility. This allows for enhanced transcription of the IFN-g gene and cytokine production through acetyl-CoA synthetase (ACSS) (168). *Ex vivo* acetate therapy also demonstrated a boost in IFN-γ production by fatigued T cells, suggesting that acetate supplementation holds the potential to reactivate hyporesponsive T cells (168). Reduced ACSS expression led to decreased IFN-γ production in tumor-infiltrating lymphocytes, hindering tumor clearance. This indicates that mechanisms governing alternative substrate use, such as acetate, could be therapeutically targeted to enhance T cell activity in cancer (168). This study unveils the capacity of acetate to rescue effector function in glucose-restricted CD8+ T cells, shedding light on potential therapeutic avenues for promoting T cell function in the context of cancer. The identified mechanisms involving histone acetylation, chromatin accessibility, and ACSS provide insights into the epigenetic remodeling of T cells by acetate. The findings suggest that targeting pathways regulating substrate utilization, particularly acetate supplementation, could be a promising strategy to enhance T cell responses and improve outcomes in cancer immunotherapy.

Ye et al. delved into the impact of chronic stress on breast cancer growth, focusing on microbiological and metabolic signals (173). The findings suggest that reduced levels of the bacterial species *Blautia* and its metabolite acetate may contribute to chronic stress-induced breast cancer growth (173). This study employed animal models to investigate the effects of Blautia and acetate therapy on CD8+ T cell responses and cancer development (173). Chronic stress and depression disrupt the gut microbiota, influencing immunological regulation and elevating the risk of colitis (174). The precise mechanisms through which microbial signals affect the tumor-associated immune response under chronic stress remain unknown. However, microbial metabolites, particularly SCFAs, significantly impact host immunity (86, 175). Ye et al. found that acetate derived from microbiota might assist in restoring T-cell immunity that has been compromised by malignancies (173). The gut microbiota’s conversion of dietary fiber into acetate has been associated with the control of allergic airway illness and hematopoiesis (176). Chronic stress correlated with decreased abundances of *Blautia* and acetate, suggesting a potential role in breast cancer growth. The study revealed that *Blautia* and acetate therapy enhanced the antitumor responses of CD8+ T cells and reversed stress-induced breast cancer growth in female mice (173).

Patients with depression exhibited lower abundances of *Blautia* and acetate. Breast cancer patients with depression displayed decreased acetate levels, reduced numbers of tumor-infiltrating CD8+ T cells, and an increased risk of metastasis (173). The study highlights the importance of the *Blautia*-acetate immunological axis in modulating the immune response to breast cancer and suggests its repression as a potential factor in chronic stress-promoted cancer progression (173). While the study identifies a correlation between reduced *Blautia* and acetate levels and breast cancer growth, the precise mechanisms by which these microbial signals influence the tumor-associated immune response under chronic stress remain unclear. The reliance on animal models, while informative, may not fully capture the complexities of human physiology and the gut microbiome’s interaction with stress and cancer. The study suggests a link between *Blautia* and acetate levels and cancer growth, but definitive causal relationships are not established, necessitating further research to confirm these findings. Ye et al. effectively highlight how chronic stress and depression can disrupt gut microbiota, subsequently influencing immunological regulation and increasing cancer risk. This connection emphasizes the importance of mental health in cancer prognosis and treatment. The therapeutic potential of *Blautia* and acetate in enhancing CD8+ T cell responses and countering stress-induced cancer progression is promising. This could lead to new interventions that incorporate microbiome modulation in cancer therapy. To translate these findings into clinical practice, studies involving human subjects are essential. Further research is needed to elucidate the precise mechanisms by which *Blautia* and acetate influence tumor-associated immune responses, particularly under chronic stress conditions.

D. Miller et al. (177) investigated the impact of inhibiting ACSS2, a crucial enzyme in acetate metabolism, on the TME and anticancer immunity. In models of *Clostridioides difficile* infection, acetate indeed enhances innate immune responses by acting on both innate-like cells and neutrophils (178). Moreover, research has demonstrated that in areas of *Listeria monocytogenes* infection, local concentrations of acetate exceed 5 mM, and effector T cells preferentially utilize acetate over glucose for ACSS (178). They also observed a time- and dose-dependent increase in Ly6C surface expression on OT-I memory T cells exposed to acetate, with Ly6C serving as a hallmark of central memory T cells, signifying its significant role in homing (179, 180). scRNA-seq analysis by D. Miller et al. revealed heightened Ly6C2 expression in CD8+ T cells, suggesting that inhibiting acetate metabolism might play a role in memory T cells (177). Additionally, histone acetylation is crucial in memory CD8+ T-cell responses (181). Acetate stimulates histone acetylation and IFN-γ synthesis by CD8+ tumor-infiltrating T cells in an ACSS -dependent manner under glucose-restricted conditions (168).

Tumor cells have been observed to dampen T-cell activity through competition for glucose, even in the presence of robust tumor antigens. This suggests that metabolic competition may suppress T-cell-driven antitumor immunity, potentially fostering cancer progression (182). ACSS2, a member of the acyl-CoA short-chain synthase family, plays a pivotal role in converting acetate in the cytoplasm and nucleus into acetyl-CoA (183). Its abundant expression in various cancers is crucial for tumor development, proliferation, invasion, and metastasis within the nutritionally stressed microenvironment. Studies have indicated that inhibitors targeting ACSS2 can effectively impede cancer development and, when combined with other antineoplastic drugs, mitigate treatment resistance (183). In the research conducted by D. Miller et al., inhibiting ACSS2 was found to transform cancer cells from acetate consumers to producers, thereby making acetate available as a fuel source for tumor-infiltrating lymphocytes. Additionally, their study revealed that acetate enhances T-cell effector functions and proliferation metabolically (177). Moreover, targeting ACSS2 using CRISPR-Cas9 guides or a small-molecule inhibitor not only hinders tumor cell metabolism but also induces an anticancer immune response, thereby enhancing the effectiveness of chemotherapy in preclinical breast cancer models (177). The study proposes a novel paradigm for targeting acetate metabolism in cancer, wherein ACSS2 inhibition serves a dual purpose of impairing tumor cell metabolism and potentiating antitumor immunity. These findings open new avenues for therapeutic interventions in breast cancer, emphasizing the interconnected roles of metabolism and immunity in cancer progression. Further exploration of ACSS2 as a potential target may lead to innovative strategies for improving the efficacy of breast cancer treatment.

In conclusion, the studies by Qiu et al. and Ye et al. illuminate the critical role of acetate in enhancing T cell function within the TME, particularly in glucose-restricted conditions prevalent in cancer. Acetate not only restores the effector function of CD8+ T cells but also facilitates metabolic adaptability through mechanisms involving histone acetylation and increased chromatin accessibility, thereby promoting cytokine production and antitumor responses. The findings suggest that therapeutic strategies aimed at modulating acetate levels or targeting the enzymes involved in acetate metabolism, such as ACSS2, could significantly improve T cell responses and overall efficacy in cancer immunotherapy. Additionally, the demonstrated link between chronic stress, reduced levels of acetate, and compromised T cell immunity underlines the importance of the gut microbiota and its metabolites in cancer progression and treatment outcomes. These insights highlight the potential for innovative therapeutic interventions that incorporate acetate supplementation or the inhibition of acetate-consuming pathways, ultimately enhancing T cell activity and improving clinical outcomes in cancer patients. However, further research, particularly in human clinical trials, is necessary to establish the mechanisms and efficacy of these approaches, paving the way for personalized and microbiome-informed cancer therapies that consider the intricate interplay between metabolism, immunity, and mental health.

### 3.3 Microbiota-derived tryptophan

Tryptophan, an essential amino acid for humans, is sourced from dietary proteins. Despite the predominant breakdown and absorption of dietary proteins in the small intestine, a notable amount of proteins and amino acids (6-18g/day) may potentially reach the colon. In the colon, beneficial bacteria play a role in breaking down these substances (184). Factors such as higher protein consumption, reduced carbohydrate levels in the colon, elevated pH in the colon, and an extended duration of passage through the colon enhance the breakdown of bacterial proteins (185–188). Several bacterial species have demonstrated the ability to convert tryptophan into indole and its derivatives (188–192). For instance, *Clostridium sporogenes* metabolizes tryptophan, producing tryptamine, ILA, and indolepropionic acid (IPA) (7, 192, 193). Indole and IAA are present in human fecal samples from healthy individuals at average quantities of 2.6 millimolar (194) and 5 micromolar (195), respectively. However, the levels of additional byproducts of tryptophan breakdown (such as IPA, ILA, IAld, tryptamine, and IA) in the human gastrointestinal tract have not been assessed to our knowledge.

Tryptophan serves various metabolic functions, including its integration into polypeptide chains of bacterial enzymes and acting as a precursor for the coenzyme nicotinamide adenine dinucleotide (196). However, within the intricate intestinal environment, the most pivotal aspect is likely the bacterial requirement for maintaining redox equilibrium. Tryptophan catabolites play crucial roles as signaling molecules and antibacterial agents (197), serving as ligands for Aryl hydrocarbon Receptor (AhR) (198–200), enhancing the activities of the intestinal epithelial barrier (201, 202), and regulating gut hormone production (203), among other functions. There is compelling evidence that tryptophan catabolites play a vital role in both maintaining good health and contributing to the development of illnesses such as IBD, neurological disorders, and cancer (204–206). This section provides current insights into the involvement of microbial-derived metabolites in cancer immunotherapy, anti-tumor immunity, and chemotherapy.

#### 3.3.1 The role of microbiota-derived tryptophan in anti-tumor immunity

Zhang et al. recently conducted a study to investigate the influence of a specific probiotic strain, *Lactobacillus plantarum L168*, and one of its byproducts, ILA, on reducing the development of colorectal tumors (24). The research commences by acknowledging previous studies that suggest the involvement of *Lactobacillus* species in diminishing CRC in mouse models. This sets the stage for the current investigation, which aims to unveil the mechanisms responsible for this phenomenon (24). The gut microbiota and its metabolites have the capacity to regulate various epigenetic processes, including DNA methylation, miRNA, RNA methylation, and histone modification (207). Currently, there is limited understanding of the precise mechanism and significance of the interaction between gut microbiota and epigenetic pathways in CRC. Previous research has demonstrated that metabolites produced by gut microbiota, including SCFAs, significantly impact immune system modulation through epigenetic mechanisms (208). Smith et al. demonstrated that the HDAC inhibitory activity of SCFAs may increase the quantity and immunosuppressive function of Treg cells (209). In contrast to SCFAs produced by intestinal bacteria, ILA does not impede the enzymatic function of HDACs in DCs to regulate the binding signals of H3K27ac. A previous study established that certain pioneering transcription factors can bind to compacted chromatin, preparing enhancers for activation. Subsequently, lineage-specific transcription factors are attracted to these primed enhancers, leading to their activation and subsequent tagging by H3K27ac (209). The research by Zhang et al. reveals that the utilization of *Lactobacillus plantarum L168* and its metabolite, ILA, results in several advantageous outcomes. These include the mitigation of intestinal inflammation, suppression of tumor proliferation, and resolution of gut dysbiosis (24). These findings suggest a potential anti-cancer influence. The researchers observed that ILA has the ability to regulate the binding effectiveness of prospective pioneer transcription factors and H3K27ac simultaneously. This implies that ILA may play a role in recruiting pioneer transcription factors to facilitate epigenetic modifications (24). Recent investigations have indicated that certain metabolites can directly interact with chromatin, thereby influencing its state or modifying the characteristics of chromatin regulatory factors (207). Consistent with these discoveries, Zhang et al. noted that ILA interacts with CTCF and influences CTCF binding at specific locations linked to reduced chromatin accessibility and enhanced binding signals of H3K27me3 (24). CTCF, a master regulator of gene expression, plays a direct role in controlling chromatin structure and maintaining the balance between active and inhibitory chromatin marks (210, 211). The Zhang et al. study delves into the mechanistic details of how ILA exerts its effects. It is shown to enhance the production of IL12a in DCs by promoting the binding of H3K27ac at the enhancer regions of IL12a. This contributes to the priming of CD8+ T cell immunity against tumor growth (24). ILA is found to transcriptionally inhibit the expression of Saa3, which is related to cholesterol metabolism in CD8+ T cells (24). This inhibition is mediated by changes in chromatin accessibility. The result is an enhanced function of tumor-infiltrating CD8+ T cells. Overall, this research offers valuable insights into the mechanisms underlying the positive effects of *Lactobacillus plantarum L168* and ILA on colorectal tumorigenesis. It points to the potential development of therapeutic strategies for CRC patients that involve probiotics and epigenetic regulation to enhance CD8+ T cell immunity against tumors (**Figure 1**, **Table 1**). These findings have implications for the development of new treatments and interventions in the field of cancer immunotherapy.

In sum, the study by Zhang et al. highlights the promising potential of the probiotic strain Lactobacillus plantarum L168 and its metabolite ILA in mitigating colorectal tumor development through multifaceted mechanisms, including the modulation of epigenetic pathways. By revealing how ILA influences chromatin dynamics and enhances CD8+ T cell immunity, this research underscores the intricate relationship between gut microbiota, metabolites, and immune responses in the context of cancer. The ability of ILA to enhance IL12a production in DCs and its impact on chromatin accessibility signify important steps toward optimizing antitumor immunity. The implications of these findings are profound, suggesting that integrating probiotics and their metabolites into cancer treatment strategies could enhance therapeutic outcomes for CRC patients. This approach may offer a novel avenue for cancer immunotherapy, particularly by leveraging the epigenetic regulatory capabilities of gut-derived metabolites. Future research should focus on clinical trials to validate these findings in human subjects and explore the broader applications of probiotic interventions in other types of cancer, ultimately contributing to personalized and effective cancer therapies.

#### 3.3.2 The role of microbiota-derived tryptophan in cancer immunotherapy

Fong et al. conducted a study on the immunomodulatory activities of Lactobacillus gallinarum and its potential role in enhancing the efficacy of anti-PD1 immunotherapy against CRC (212). It has been established that the recruitment and infiltration of Treg cells are major contributors to immune checkpoint blockade (ICB) resistance, creating an immunosuppressive TME that hinders the cytotoxicity of effector T cells (213). They initially demonstrated that *Lactobacillus gallinarum* significantly enhanced the effectiveness of anti-PD1 and led to substantial tumor shrinkage (212). Emerging evidence suggests that the composition of the gut microbiota plays a pivotal role in the success of cancer immunotherapy, and modifying the microbiota is considered a potential strategy to enhance ICB response (212). When combined with anti-PD1, *Lactobacillus gallinarum* reduced the infiltration of Foxp3+CD25+ Tregs in the TME and increased the effector activity of CD8+ T cells, indicating improved antitumor immunity. Remarkably, *Lactobacillus gallinarum* enhanced the effectiveness of anti-PD1 in the CT26 syngeneic mouse model, characterized by MSI-low status and known for its poor response to immunotherapy (212). The identified functional metabolite responsible for these observed effects is indole-3-carboxylic acid (ICA), produced by *Lactobacillus gallinarum* (212). A recent study found that the probiotic *Lactobacillus acidophilus* enhances ICB efficiency by reducing intratumoral Tregs and increasing effector CD8+ T cells (214).

The indole pathway, both in the host and bacterium, is renowned for its highly dynamic nature, involving the rapid conversion and breakdown of various indole metabolites in *in-vivo* (215). ICA is directly derived from IAld, requiring only a single oxidation step for the conversion from IAld to ICA. It has been hypothesized that this conversion is facilitated by cytochrome P450 enzymes in the liver, which are known for their involvement in the transformation of indole metabolites *in vivo* (216, 217).

Indoleamine 2,3-dioxygenase 1 (IDO1), a widely expressed enzyme in various human malignancies, plays a crucial role in the conversion of tryptophan to kynurenine (Kyn) and has been identified as a promising target for pharmacological intervention (218). Several studies have highlighted the ability of probiotics to suppress IDO1 expression and reduce Kyn levels in living organisms (219, 220). The Kyn/Trp ratio is acknowledged as a prognostic marker for predicting the response to ICB (221). In their study, Fong et al. demonstrated that ICA inhibited the activity of IDO1, resulting in a decrease in Kyn synthesis in tumors (212). Moreover, their findings indicated that ICA and Kyn competed for the same binding site on the AHR, with ICA impeding Kyn’s binding to CD4+ T cells (212). Consequently, Treg cell development was suppressed under laboratory conditions. Numerous investigations have highlighted the antagonistic effects of indole metabolites, which, while modestly activating the AHR themselves, also exhibit antagonistic activity by inhibiting AHR activation induced by agonists (222). Recent discoveries suggest that indole metabolites produced by *Lactobacillus* induce immunosuppression by activating the AHR on tumor-associated macrophages, thereby fostering tumor growth (223). The investigation conducted by Fong et al. revealed a competitive interaction between AHR ligands originating from the host and the microbiome. Specifically, they observed that ICA, originating from *Lactobacillus gallinarum* (a weak AHR agonist), surpassed Kyn and impeded Kyn-induced AHR activation. The use of partial agonists is a common strategy in drug development (212). Their results showed that administering ICA replicated the effects of *Lactobacillus gallinarum* and significantly improved the efficacy of anti-PD1 treatment in live organisms (212). However, supplementing with Kyn might counteract the enhanced effectiveness (212). Through the modulation of the IDO1/Kyn/AHR axis using ICA, *Lactobacillus gallinarum* has emerged as a potential adjunct to augment the effectiveness of anti-PD1 immunotherapy against CRC.

The study demonstrates that *Lactobacillus gallinarum* can enhance the response to anti-PD1 therapy in CRC through its immunomodulatory effects. The identified metabolite, ICA, plays a crucial role in suppressing Treg differentiation and enhancing the function of CD8+ T cells. The mechanistic insights into the IDO1/Kyn/AHR axis provide a clear understanding of how *Lactobacillus gallinarum*-derived metabolites exert their effects. The findings suggest a promising avenue for the development of microbial-based adjuvants to improve the efficacy of immunotherapy in CRC. *Lactobacillus gallinarum* and its metabolite ICA could potentially be integrated into therapeutic strategies for CRC treatment. The study contributes valuable knowledge to the evolving field of microbiota-mediated immunomodulation and its implications for cancer therapy.

In conclusion, the study by Fong et al. reveals the significant role of *Lactobacillus gallinarum* and its metabolite ICA in enhancing the efficacy of anti-PD1 immunotherapy against CRC. By demonstrating that Lactobacillus gallinarum reduces Treg infiltration and increases CD8+ T cell activity, the research highlights a novel immunomodulatory mechanism that could combat resistance to ICB. The mechanistic insights regarding the IDO1/Kyn/AHR axis provide a deeper understanding of how Lactobacillus-derived metabolites influence immune responses, with ICA acting as a competitive antagonist to Kyn and suppressing Treg cell differentiation. These findings suggesting that *Lactobacillus gallinarum* could serve as a valuable adjunct therapy in CRC treatment, particularly for patients who exhibit poor responses to current immunotherapies. This research supports the potential of microbiome modulation as a strategy to enhance cancer treatment outcomes. Future studies are warranted to explore the clinical applications of Lactobacillus gallinarum and ICA in CRC patients and other malignancies, as well as to further investigate the broader impact of gut microbiota on the efficacy of immunotherapeutic strategies in oncology. Integrating probiotics into cancer care could pave the way for innovative therapeutic approaches that harness the power of the microbiome to improve patient outcomes.

#### 3.3.3 The role of microbiota-derived tryptophan in chemotherapy

**Figure 2:**
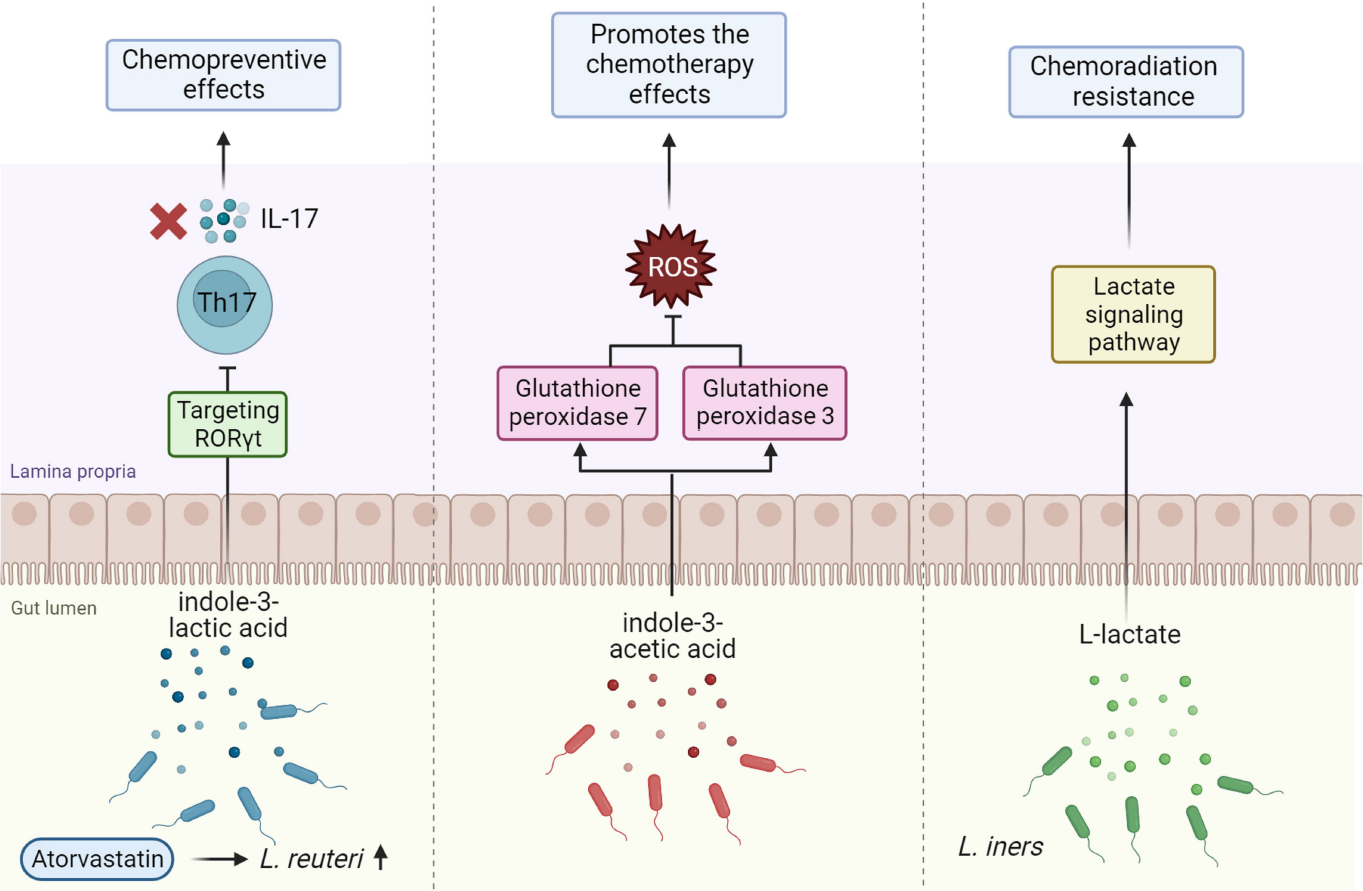
The roles of gut microbiota-derived metabolites in modulating cancer chemotherapy. Indole-3-lactic acid produced by *Lactobacillus reuteri*, especially when stimulated by atorvastatin, exhibits chemopreventive effects by targeting the RAR-related orphan receptor γt (RORγt) and inhibiting the production of IL-17 from Th17 cells. Indole-3-acetic acid enhances the effectiveness of chemotherapy by downregulating reactive oxygen species (ROS)-degrading enzymes such as glutathione peroxidase 3 and 7, thus increasing ROS levels which promote chemotherapy-induced cytotoxicity. Additionally, L-lactate produced by *Lactobacillus iners* contributes to chemoradiation resistance through the activation of lactate signaling pathways. This figure highlights the complex interplay between gut microbiota metabolites and cancer treatment modalities.

Tintelnot et al. delved into the impact of microbiota-derived 3-indoleacetic acid (3-IAA) on chemotherapy efficacy in pancreatic ductal adenocarcinoma (PDAC) (**Figure 2**, **Table 1**) (35). PDAC, known for its exceptional aggressiveness and often unfavorable prognosis due to limited treatment efficacy, prompted the investigation into the potential influence of nutrition and gut microbiota on treatment outcomes. Recognizing that conventional genetic explanations alone fall short in explaining the variable responses of individuals to chemotherapy in PDAC, the study aimed to explore the role of microbiota and dietary patterns in treatment outcomes (35). In a subset of individuals with localized PDAC who exhibited prolonged survival, the migration of bacteria from the gut to the tumor was observed, regulating the activation of the immune system against the tumor. However, for most patients with advanced, immunotherapy-resistant PDAC, combination chemotherapy remains a common treatment approach, and the impact of microbiota or dietary patterns on this treatment remains unclear (224–227). The research conducted by Tintelnot et al. revealed a possible link between positive chemotherapy outcomes and increased levels of 3-IAA, a byproduct of gut bacteria that is produced through the breakdown of tryptophan (35). To further investigate, the researchers conducted studies using humanized gnotobiotic mouse models of PDAC. Their findings indicated that fecal microbiota transplantation, short-term dietary modifications of tryptophan, and oral administration of 3-IAA all contributed to enhanced chemotherapy effectiveness (35).

This suggests that modifying the gut microbiota or supplementing with 3-IAA could represent strategies to enhance the efficacy of PDAC treatment. The study elucidates that the synergy between 3-IAA and chemotherapy relies on the activity of myeloperoxidase produced by neutrophils (35). Myeloperoxidase catalyzes the oxidation of 3-IAA, and when used in conjunction with chemotherapy, it results in the reduction of enzymes responsible for breaking down ROS (35). Consequently, this leads to the accumulation of ROS and the suppression of autophagy in cancer cells (35). These alterations compromise the metabolic efficiency of cancer cells, ultimately impeding their growth (35). Moreover, the study highlights a significant association between the levels of 3-IAA and treatment effectiveness in two separate cohorts of PDAC patients (35). This suggests that the detection of 3-IAA in patients might potentially function as a prognostic indicator for their treatment outcome. The findings of this research are significant because they shed light on an area of cancer therapy that has been previously overlooked: the role of the gastrointestinal microbiota and its metabolites, specifically 3-IAA.

The study presents novel therapeutic interventions by elucidating the underlying mechanisms and demonstrating that the presence of 3-IAA in the intestinal microbiota is associated with improved responses to chemotherapy in PDAC. These interventions could involve manipulating the gut microbiota, altering dietary patterns, or directly administering 3-IAA as a complement to traditional chemotherapy. Moreover, the study emphasizes the importance of considering the influence of diet and the gut microbiota on cancer treatment outcomes, highlighting the need for a holistic approach to cancer therapy that includes nutritional interventions. These findings have broader implications for personalized medicine, where treatment strategies can be tailored to individual patients based on their gut microbiota composition and the presence of specific metabolites like 3-IAA. This research may ultimately lead to improved treatment regimens and more favorable outcomes for patients with PDAC.

Han et al. investigated the chemopreventive effects of tryptophan catabolites produced by the microbiota in individuals with CRC (37). CRC is highly preventable, typically developing slowly through a series of changes from adenoma to carcinoma (228). Clinical research has been concentrated on exploring the chemopreventive impact of statins in CRC (229–232). The study begins by acknowledging prior epidemiological investigations that have indicated a correlation between the use of statins and a reduced incidence of CRC (37). Statins, a class of medications commonly used to lower cholesterol levels, have also shown promise in preventing CRC (37). Through retrospective studies on individuals who underwent colonoscopies, the researchers uncovered evidence supporting the chemopreventive role of statins in reducing the occurrence of CRC (37). This finding strengthens the feasibility of employing statins for the prevention of CRC. Previous studies have already demonstrated the role of bacteria in influencing the efficacy of drugs (233–235). Additionally, a study conducted by Han et al. demonstrated that the mitigating effect of statin therapy on tumor size was associated with its influence on the gastrointestinal microbiota. The use of statins led to changes in the composition of the gut microbiota, particularly an increase in the presence of the beneficial bacteria *Lactobacillus reuteri* (*L. reuteri*) (37). The heightened prevalence of *L. reuteri* was associated with the increased availability of tryptophan in the gut microbiome following treatment with atorvastatin, a specific type of statin medication (37). These findings suggest that statins contribute to elevating the levels of tryptophan in the gut microbiota, thereby influencing the microbial composition. The beneficial effects of *L. reuteri* may be attributed to its ability to combat microorganisms and regulate the immune system. A previous study demonstrated that reuterin, one of the antimicrobial compounds produced by *L. reuteri*, suppressed the proliferation of CRC cells by promoting protein oxidation and inhibiting ribosome biogenesis (236). *In vivo* investigations by Han et al. revealed that the administration of *L. reuteri* resulted in the synthesis of ILA, a catabolite of tryptophan (37). They showed that ILA, a compound produced by *L. reuteri*, is an additional crucial factor in inhibiting the onset of CRC (37). Notably, a prior study demonstrated that ILA produced by *Lactobacillus gallinarum* induced apoptosis in CRC cell lines (237). The role of IL-17A in promoting the development of intestinal tumors has been extensively studied, and the removal of IL-17A has a significant impact in reducing the number of tumors in ApcMin mice (238, 239). Previous studies have demonstrated that oncomicrobes enhance the formation of CRC by activating the IL-17 signaling pathway (240, 241). According to the findings of Han et al., ILA exhibited anti-tumorigenic effects by downregulating the IL-17 signaling pathway, which is associated with inflammation and immunological responses (37). ILA reduced the development of T helper 17 cells by targeting the nuclear receptor, RAR-related orphan receptor t (RORt). The research by Han et al. (37) illustrated that *L. reuteri* inhibits CRC carcinogenesis by blocking the IL-17 signaling pathway. The AHR operates downstream of indole derivatives to help cells adapt to environmental changes (242). Their results indicate that activating AHR in the epithelium enhances barrier integrity in ApcMin mice, contributing to ILA’s overall anti-cancer impact (**Figure 2**, **Table 1**) (37). The research provides detailed mechanistic insights into how statins might influence CRC prevention, particularly through their impact on gut microbiota and tryptophan metabolism. Findings from animal models, such as ApcMin mice, may not fully translate to human physiology and cancer development, potentially limiting the applicability of the results. The emphasis on Lactobacillus reuteri and its metabolites may overlook other significant microbial players and metabolites that could contribute to CRC prevention. To validate the chemopreventive potential of statins and the role of gut microbiota in CRC prevention, prospective clinical trials are essential. Expanding the scope to include other microbial species and metabolites could provide a more comprehensive understanding of the gut microbiome’s role in CRC prevention. Finally, translating these findings from animal models to human studies will be crucial in determining the clinical applicability and potential therapeutic strategies involving gut microbiota modulation.

In conclusion, the studies by Tintelnot et al. and Han et al. underscore the vital role of gut microbiota and its metabolites in enhancing chemotherapy efficacy and preventing CRC, respectively. Tintelnot et al. demonstrated that the microbiota-derived metabolite 3-IAA correlates with improved chemotherapy outcomes in pancreatic ductal PDAC. Their findings suggest that modifying gut microbiota or supplementing with 3-IAA could serve as promising strategies to enhance the effectiveness of PDAC treatment. Similarly, Han et al. revealed how the probiotic Lactobacillus reuteri and its metabolites can influence CRC prevention through mechanisms involving tryptophan metabolism and immune modulation. The research highlights the chemopreventive potential of statins, particularly in how they alter gut microbiota composition to increase beneficial bacteria like *L. reuteri*. The implications of these findings are significant, indicating that a holistic approach that incorporates dietary patterns and gut microbiota manipulation could improve cancer treatment outcomes. For PDAC patients, incorporating 3-IAA as a dietary supplement or through microbiota modulation might enhance chemotherapy responses. In CRC prevention, leveraging the benefits of probiotics such as *L. reuteri* alongside statin therapy could represent a novel and effective strategy. Furthermore, the studies emphasize the need for personalized medicine, where treatment plans could be tailored based on an individual’s gut microbiota composition and the metabolites present. Future research should focus on validating these findings in clinical settings and exploring the broader implications of microbiota interactions in cancer therapy. Prospective clinical trials are necessary to confirm the efficacy of these interventions in human populations and to assess their long-term impact on treatment outcomes. By bridging the gap between basic research and clinical application, these insights could lead to innovative therapeutic strategies that harness the power of the gut microbiome to combat cancer effectively.

### 3.4 TMAO

TMAO is a small chemical molecule with a molecular mass of 75.1 Daltons, belonging to the amine oxide family. It is commonly present in the tissues of various marine creatures (243). TMAO levels increase in the serum of animals colonized with TMA-producing bacteria, in contrast to animals colonized with bacteria incapable of producing TMA from choline *in vitro* (244). Primarily generated by intestinal bacteria in the colon from dietary substrates such as phosphatidylcholine/choline, carnitine, betaine, dimethylglycine, and ergothioneine, TMA is absorbed into the circulation and converted to TMAO by hepatic flavin monooxygenases (FMO1 and FMO3). Alternatively, it may undergo metabolism in the colon to methylamine, dimethylamine (DMA), and ammonia (245–247). Choline-rich foods such as red meat, eggs, dairy products, and saltwater fish are potential sources of TMAO. TMA, initially a gas, undergoes oxidation within living organisms by FMO1 and FMO3 to produce TMAO (248, 249). Subsequently, TMAO is either transported to tissues for osmolyte accumulation or, more commonly, excreted by the kidneys (54, 250, 251). Numerous factors, including age, gender, diet, composition of intestinal microbiota, renal function, and liver FMO activity, influence TMAO levels (12). Numerous studies have established connections between TMAO levels and the onset of various conditions, including cancer (252–254), cardiovascular disease (255, 256), metabolic syndrome (257), chronic renal disease (258), and diabetes mellitus (259). In this segment, our focus is on examining TMAO’s role in anti-tumor immunity, cancer immunotherapy, and chemotherapy.

#### 3.4.1 The role of TMAO in anti-tumor immunity

Wang et al. (260) delved into the role of the microbial metabolite TMAO in modulating the TME and its impact on the outcomes of immunotherapy in triple-negative breast cancer (TNBC). Initially, commensal microbiota was considered pathogens and implicated in certain cancers, like Helicobacter pylori in gastric cancer (261, 262). Recent research has shifted focus towards understanding the interactions between commensal microbiota and the TME, with the majority of investigations conducted in mice and yielding conflicting findings (224, 263–266). Wang et al. carried out a comprehensive multiomics analysis involving a cohort of 360 TNBC patients, revealing a higher abundance of *Clostridiales* and TMAO genera in tumors with an active immune microenvironment (260). Additionally, individuals with elevated plasma TMAO levels exhibited a more favorable response to immunotherapy (260). The study identified that Clostridiales genera and the metabolite TMAO were more prevalent in tumors with an active immune microenvironment (260). Patients with increased plasma TMAO levels demonstrated better responses to immunotherapy, suggesting a potential predictive biomarker for therapy outcomes (260). However, due to challenges in colonization and the associated risk of infection, fecal microbiota transplantation and oral probiotics are not currently deemed safe or viable therapies for TNBC (267). However, TMAO and its precursor, choline, may hold promise as clinical therapies. TMAO, a product of microbial metabolism, plays a role in the pathogenesis of various inflammation-related diseases, including renal and cardiac injuries (53, 56, 250, 268). Surprisingly, Wang et al. discovered that elevated plasma TMAO levels were associated with positive therapeutic effects in TNBC (260). While prior studies mainly concentrated on the direct impact of microbial metabolites on the host’s immune cells, whether these metabolites can modulate the function of tumor cells remained unclear (22, 23). Their findings revealed that TMAO induces pyroptosis in tumor cells by activating the endoplasmic reticulum stress kinase PERK (260). Consequently, this process enhances CD8+ T cell-mediated antitumor immunity in TNBC. The study proposes that microbial metabolites, like TMAO or its precursor choline, could present a novel therapeutic strategy to augment the effectiveness of immunotherapy in TNBC. In conclusion, this study introduces a fresh perspective on the capacity of commensal microbiota to influence host tumor immunity, suggesting that the microbial metabolite TMAO may hold clinical potential for enhancing immunotherapy efficacy in TNBC. Moreover, this study underscores the importance of considering the interactions between commensal microbiota and the TME in cancer therapy. The positive association between TMAO and immune activity highlights the need for further research into the mechanisms by which microbial metabolites influence tumor immunity. Future studies could explore the clinical application of TMAO as a therapeutic adjunct in immunotherapy regimens, potentially leading to more personalized and effective treatment strategies for TNBC patients. This research paves the way for a better understanding of how microbiota-derived metabolites can shape immune responses in cancer, ultimately contributing to improved patient outcomes.

#### 3.4.2 The role of TAMO in cancer immunotherapy

The study by Mirji et al. investigates the role of TMAO, a metabolite derived from the gut microbiome, in influencing immune responses and the effectiveness of ICB therapy in PDAC (269). The research identifies TMAO as a metabolite produced by gut microbes that may enhance the anti-tumor immune response against PDAC (269). In mice with orthotopic PDAC, TMAO is associated with reduced tumor growth (269). Additionally, they observed that TMAO administration was linked to the development of an immunostimulatory tumor-associated macrophages (TAM) phenotype, correlated with heightened anti-tumor immunity (269). Moreover, TMAO was found to stimulate effector T cell responses within the TME (269). Consistent with previous research (268, 270), TMAO was identified as a significant inducer of inflammatory effects. The findings suggest that TMAO activates the IFN pathway, crucial for antiviral and anti-tumor immune responses (269). TMAO’s anti-tumor properties were found to depend on type-I IFN signaling. These inflammatory drivers are known to influence immune cell activity, potentially regulating tumor development and therapeutic response. For instance, type-I IFN stimulation has been demonstrated to restrict TAM production while promoting polarization toward an immunostimulatory phenotype (271, 272).

Moreover, Mirji et al. made the significant observation that the combination of TMAO with immune checkpoint blockade (anti-PD1 and/or anti-Tim3) led to a substantial reduction in tumor burden and improved survival in a mouse model of PDAC, surpassing the outcomes achieved with TMAO or ICB therapy alone (269). This suggests that TMAO has the potential to enhance the effectiveness of ICB treatment. Additionally, the presence of bacteria carrying CutC, an enzyme involved in trimethylamine production (the precursor to TMAO), was found to be correlated with prolonged survival in PDAC patients. Moreover, PDAC patients with a higher abundance of these bacteria exhibited more favorable responses to anti-PD1 treatment in melanoma patients (269). The connection between the presence of bacteria harboring the CutC enzyme and improved outcomes in PDAC and melanoma patients underscores the therapeutic significance of this discovery. This suggests that approaches targeting TMAO or similar pathways may hold therapeutic promise in cancer treatment that extends beyond experimental mouse models. In summary, our research identifies TMAO as a crucial driver of anti-tumor immunity and provides evidence of its therapeutic potential in PDAC. The findings open up new avenues for exploring the role of the gut microbiome in cancer immunotherapy and suggest that targeting TMAO-related pathways may hold promise for enhancing treatment outcomes in patients with PDAC and other cancers. These findings pave the way for new therapeutic strategies that utilize microbiome-derived metabolites, such as TMAO, to enhance the effectiveness of immunotherapy in PDAC and potentially other malignancies. Future research could investigate the clinical applicability of targeting TMAO-related pathways and the role of the gut microbiome in modulating tumor immunity. This study not only provides valuable insights into the intersection of microbiome health and cancer therapy but also suggests pathways for developing personalized treatment approaches that harness the body’s own immune mechanisms.

### 3.5 Urolithin A

Urolithins, a group of chemical molecules characterized by benzo-coumarin scaffolds, represent ellagitannin and ellagic acid metabolites produced by the gut microbiota (273, 274). UA is a naturally occurring molecule synthesized by gut bacteria from ingested ellagitannins (ETs) and ellagic acid (EA), both complex polyphenols present in foods like pomegranates, berries, and nuts (275). A groundbreaking study has also revealed that the human gut microbiota synthesizes UA from ETs (276), establishing UA as the most prevalent urolithin species produced in nature. The effects of UA have been documented across a diverse range of health conditions in both *in vitro* and *in vivo* models. The metabolite has demonstrated anti-inflammatory, anti-apoptotic, and antioxidant properties, along with cardioprotective and neuroprotective qualities (275). Despite promising *in vivo* studies, conclusive evidence regarding UA’s health benefits and mechanisms of action is still a topic of debate. Recent research has highlighted the significant role of UA in cancer pathobiology (277–281). For instance, Cheng et al. investigated the impact of UA, a metabolite found in pomegranate ellagitannins, on the epithelial-to-mesenchymal transition (EMT) in lung cancer cells. Urolithin A effectively impeded the EMT process, evident through the upregulation of epithelial markers and the inhibition of mesenchymal markers, according to their findings. This inhibitory effect was achieved by disrupting the interaction between p53 and Mdm2, leading to the ubiquitination and degradation of Snail, a critical regulator of EMT (277).

Norden et al. conducted a study to investigate the impact of various polyphenolic metabolites produced by gut microbes, including UA, on the proliferation of colon cancer cells. They also explored the interaction of these metabolites with oxaliplatin, a chemotherapy drug (280). UA, among the metabolites, exhibited a significant inhibitory effect on cell proliferation, displaying an IC50 value of 19 µM. Furthermore, it demonstrated a synergistic effect when combined with oxaliplatin. The researchers observed that UA stabilized p53 and increased the expression of genes targeted by p53. Importantly, the antiproliferative effect of UA was markedly reduced in the absence of p53. This suggests the essential role of the p53/TIGAR axis in mediating the effects of UA, leading to a reduction in glycolytic potential. These findings underscore the potential of UA as a dietary chemopreventive agent in colon cancer (280). Moreover, a recent analysis has indicated that UA plays a crucial role in the effectiveness of cancer immunotherapy and the body’s ability to combat tumors (281–284).

#### 3.5.1 The role of UA in anti-tumor immunity

Denk et al. delved into the proliferation of T memory stem cells (TSCM) and their enhanced immune response against tumors by inducing mitophagy through the use of UA (282). TSCM cells are thought to fortify the immune system by consistently generating less fatigued effector T cells within the TME (282). T cell fate decisions are expected to be regulated by various mechanisms that maintain a balance between stemness and final differentiation (285–287). The authors propose a complex interaction between internal signals from the mitochondria and the subsequent activation of the stem cell-promoting Wnt-driven Tcf7 gene transcription (282). The impact of UA on TSCM development appears to depend on both Pink1 and Pgam5. Notably, Pgam5 is suggested to govern the Pink1/Parkin-mediated mitophagy process (288). Initial evidence suggests that PGC-1α is a crucial regulator directly stimulated by Wnt signaling, playing a vital role in TSCM establishment. This hypothesis has been proposed in the context of Mek inhibition (282). Previous research has demonstrated that both pomegranate juice and pure elagitannins exhibit antiproliferative and apoptotic effects in CRC cell lines when tested *in-vitro* (289, 290). The results highlight that UA, a substance synthesized by the gut microbiota from ellagitannin-rich meals, holds the potential to augment the population of TSCM cells (282). This discovery suggests a potential strategy to enhance the body’s intrinsic anti-tumor mechanisms. Denk et al. observed that when tumor-bearing mice were orally administered UA, they exhibited a robust CD8+ T cell response against the tumor (282). This indicates that UA has the capacity to enhance the immune response against malignancies in living organisms. The research conducted by Denk et al. also revealed that T cells, pre-treated with UA in a controlled *in vitro* setting, exhibited enhanced anti-tumor capabilities when subsequently utilized in adoptive cell transfer (282). This implies that UA has the potential to boost the efficacy of immune cell treatments, a common approach in cancer treatment. Furthermore, it was demonstrated that the creation of UA-induced TSCM is contingent upon Pink1-mediated mitophagy, a mechanism aiding in the removal of impaired mitochondria (282). Therefore, it may be inferred that mitophagy plays a pivotal role in the proliferation of TSCM cells and, consequently, in enhancing the immune response against tumors. Their findings showed that Pgam5, a phosphatase located in the mitochondria, is released into the cytosol during the process of mitophagy. These results indicate that Pgam5 may play a role in triggering the establishment of TSCM (282). The Wnt-β-Catenin signaling pathway is suggested to stimulate the process of mitochondrial biogenesis (291). The present investigation revealed that cytosolic Pgam5 has the ability to dephosphorylate β-catenin, a protein involved in Wnt signaling, a crucial pathway for cellular proliferation and differentiation (282). The results suggest that the dephosphorylation of β-catenin initiates the activation of Wnt signaling. Additionally, the findings indicate that Wnt signaling initiates a compensatory process of mitochondrial biogenesis. This process may serve as a mechanism through which UA-induced TSCM cells proliferate and flourish (282). In summary, this study unveils a crucial signaling mechanism in which mitophagy, triggered by UA, leads to the generation of TSCM, enhancing the body’s immune response against tumors. The results suggest that UA, a metabolically well-tolerated drug, could be a potentially beneficial option for enhancing immune therapy in the context of cancer treatment. The findings of this study may hold substantial significance for the development of innovative immunotherapies and strategies to augment the innate tumor-fighting mechanisms of the body.

Ginefra et al. recently undertook a study investigating the impact of UA on CD8+ T cells and their role in cancer immunosurveillance (292). CD8+ T cells play a pivotal role in the immune system’s defense against infections and cancer (293–295). The aging process and the onset of cancer can lead to deficiencies in both the quantity and effectiveness of functional naïve T cells, impeding the immune system’s ability to monitor and combat diseases. Interventions are needed to enhance the quality and quantity of these T cells for cancer prevention through immunotherapy (292). The study examines the direct influence of UA on the balance and effectiveness of CD8+ T lymphocytes. It demonstrates that orally administered UA inhibits tumor progression in mice, and this outcome is dependent on the presence of CD8+ T cells (292). This suggests that UA enhances the immune system’s ability to detect and eliminate cancer cells. Furthermore, UA supplementation was found to induce the proliferation of naïve CD8+ T lymphocytes *in vivo* (292). This is significant as naïve T cells are essential for a robust immunological response. UA amplifies cytokine production and mitochondrial activity in CD8+ T lymphocytes (292). This indicates that UA enhances the functionality of these cells, thereby increasing their efficacy in fighting against cancer. UA also promotes memory specialization in CD8+ T cells *in vitro* (292). Memory T cells play a crucial role in sustaining long-lasting immunity by retaining information from prior interactions with infections or cancer cells. The study reveals that the transcription factor FOXO1 is a target of UA in CD8+ T lymphocytes, independent of mitochondria (292). Consequently, it can be inferred that UA operates through a mechanism not reliant on mitophagy but instead involves the stimulation of FOXO1. The research aims to determine the most effective dose and timing of UA supplementation *in vitro* to induce prolonged growth of CD8+ T cells with enhanced anti-tumor properties, particularly in the context of adoptive T cell therapy (292). The study’s findings offer preclinical data supporting the use of UA as a novel immunomodulator to enhance immunosurveillance. UA enhances the efficacy of CD8+ T lymphocytes in combating cancer by improving both their quality and quantity. This study suggests that UA may possess therapeutic properties as an immunomodulatory drug, potentially enhancing the body’s ability to recognize and combat cancer. Overall, these findings offer substantial preclinical evidence supporting the use of UA as a novel immunomodulator to enhance cancer immunosurveillance and improve the body’s ability to combat cancer. UA’s well-tolerated nature and its potential to enhance immune therapy hold promise for the development of novel immunotherapies and strategies that could significantly benefit cancer prevention and treatment. This research underscores the importance of further investigation into UA’s role in cancer immunotherapy and its potential clinical applications.

In conclusion, the studies conducted by Denk et al. and Ginefra et al. reveal the potential of UA as a powerful immunomodulator in enhancing the immune response against tumors, particularly in the context of TSCM and CD8+ T lymphocytes. Denk et al. demonstrated that UA promotes the proliferation of TSCM through mitophagy, which in turn enhances the anti-tumor immune response. Their findings indicate that UA’s impact on TSCM development is linked to key signaling pathways, such as the Pink1/Parkin-mediated mitophagy and Wnt-β-catenin signaling, suggesting a complex interplay between mitochondrial function and immune cell dynamics. Additionally, the ability of UA to improve the efficacy of adoptive T cell transfer underscores its therapeutic potential. Similarly, Ginefra et al. highlighted the role of UA in augmenting CD8+ T cell functionality, promoting their proliferation and enhancing cytokine production. By supporting the development of memory T cells, UA may foster long-lasting immunity against cancer. The research indicates that UA acts through mechanisms that are both dependent and independent of mitophagy, specifically targeting transcription factors like FOXO1 to enhance T cell effectiveness. Together, these findings suggest that UA may serve as a promising therapeutic agent for improving cancer immunotherapy outcomes. Its well-tolerated nature and ability to bolster the immune system’s innate capabilities could lead to innovative treatment strategies that enhance both the quality and quantity of anti-tumor immune responses. Future research should focus on optimizing UA dosing and administration timing to maximize its benefits in clinical settings, paving the way for new approaches to cancer prevention and treatment that leverage the body’s own immune mechanisms.

#### 3.5.2 The role of urolithin in cancer immunotherapy

The research by Wang et al. delves into the therapeutic potential of urolithin B (UB), a metabolite originating from polyphenols within the gastrointestinal tract, aiming to prevent CRC and enhance the immune microenvironment in CRC (284). Their investigation reveals that UB exerts a preventive effect on CRC development in murine CRC models, irrespective of whether induced by inflammation or genetic mutations (284). This suggests that UB may have the capacity to reduce the risk of CRC occurrence. The presence of intestinal flora in the TME of colon cancer is linked to the immune response. CRC exhibits low immunogenicity, and the prevalence of pathogenic intestinal bacteria increases following colon cancer surgery and chemotherapy (141, 296–298). The immune response mediated by the gut microbiota plays a crucial regulatory role in immunotherapy. Studies indicate that *Akkermansia muciniphila* is an immunomodulatory bacterium (296, 299). Disruptions in microbial structure and the depletion of specific bacteria impede the efficacy of immunotherapy. By comparing the intestinal flora of responders and non-responders, researchers observed a correlation between the relative abundance of *Akkermansia muciniphila* in tumor patients and the immune response. Notably, the abundance of *Akkermansia muciniphila* increased in patients who positively responded to immunotherapy, as documented in studies (296, 299). It is noteworthy that *Akkermansia muciniphila* can enhance the immune response to chemotherapeutic drugs (297, 299). The study carried out by Wang et al. revealed that the administration of UB induced changes in the composition of the gut microbiota (284). These alterations might have played a role in the reported anticancer and immunomodulatory effects. UB was associated with modifications in the tumor immunological microenvironment. Specifically, it was found to influence the expression of HLA-B, a protein involved in presenting antigens, as well as various types of immune cells, including NK cells, regulatory T cells, and γδ TCR cells (284). These changes suggest that UB has the potential to establish a more favorable immunological microenvironment for treating CRC. *Akkermansia muciniphila* is strongly linked to positive responses to anti-PD-1 treatment (300). As per the research conducted by Wang et al., the administration of UB led to a decrease in the production of Programmed death-ligand 1 (PD-L1), a protein utilized by cancer cells to evade detection by the immune system (284). Reducing PD-L1 levels holds the potential to fortify the immune response against CRC. The combination of UB with first-line treatment medications yielded improved outcomes concerning colorectal intestinal hematochezia, characterized by the presence of blood in the stool (284). This suggests that UB may enhance the effectiveness of current CRC therapies. The study indicates that UB, particularly when used in conjunction with an anti-PD-1 antibody, can hinder the proliferation of CRC. This finding implies that UB could serve not only as a preventive measure but also as an adjunct to boost the effectiveness of immunotherapy in CRC patients. In summary, Wang et al. demonstrates that UB can prevent the development of CRC, modify the gut microbiota composition, influence the immunological milieu of tumors, and reduce the expression of PD-L1. These collective factors contribute to the anticancer and immunomodulatory effects of UB. Employing UB alongside primary therapeutic medications and immunotherapy agents holds promise as an approach to enhance CRC treatment. These findings suggest that UB might significantly contribute to improving the prognosis for CRC patients, offering novel avenues for CRC therapy and prevention. However, further investigation and rigorous clinical studies are necessary to validate these promising results.

Mehra et al. explore the potential of UA, a gut microbe-derived metabolite found in pomegranates, to enhance the durability and effectiveness of ICB treatment in the context of PDAC (283). PDAC stands out as an exceptionally aggressive cancer, boasting a mere 12% 5-year survival rate and ranking among the most formidable solid organ malignancies (301). Current treatment options for PDAC are limited, with curative surgical removal feasible in only about 15%–20% of cases, primarily due to advanced disease upon diagnosis (301). Despite its transformative impact on other cancer types, immunotherapy has demonstrated limited efficacy in generating enduring responses in most PDAC patients (302–304). Uro A exhibits the capability to selectively target and inhibit specific signaling pathways implicated in PDAC, including the KRAS-dependent PI3K/AKT/mTOR pathways, aiming to overcome therapy resistance (305). However, the impact of Uro A on the tumor’s immunological environment and its potential to enhance ICB effectiveness have not been explored prior to this study. Investigations focusing on unraveling the crucial factors contributing to PDAC resistance to traditional chemotherapy and immunotherapy have revealed the pivotal role of the TME in enabling cancer cells to evade immune system detection. This, in turn, facilitates tumor growth and metastasis (306). The TME of PDAC is characterized by extensive stromal desmoplasia, a surplus of extracellular matrix proteins, immunosuppressive cellular populations, and notably deficient activated T cells. Previous research has established that this resistance trait relies on oncogenic KRAS alterations driving disease progression (307, 308). A study carried out by Mehra et al. demonstrated that UA treatment caused a restructuring of the TME in PDAC (283). This restructuring involved a reduction in stromal fibrosis, a hallmark often associated with PDAC that may impede the access of immune cells and therapies to the tumor (283). This modification is noteworthy as it holds the potential to enhance immune cell infiltration into the tumor. The study demonstrated that UA therapy effectively reinvigorated the adaptive T-cell immune response within the tumor (283). Specifically, there was an increase in the infiltration of CD4+ and CD8+ T cells displaying memory-like behavior (283). Memory T cells are recognized for their ability to elicit a more potent and enduring immune response. UA therapy led to a decrease in the expression of the protein PD-1, associated with immune exhaustion, on tumor-infiltrating immune cells (283). PD-1 serves as a crucial marker of immunological exhaustion, and its decrease signifies heightened activation and functionality of immune cells. Mehra et al. illustrated that combining UA treatment with anti-PD-1 immunotherapy resulted in a substantial enhancement of the antitumor response (283). This combination therapy led to an increased infiltration of CD4+ Th1 cells, which are known for their role in promoting antitumor immunity. The combination of UA treatment with anti-PD-1 immunotherapy resulted in a remarkable improvement in overall survival in a genetically engineered mouse model of PDAC (283). This finding suggests that UA has the potential to sensitize PDAC to immunotherapy and significantly extend survival. In summary, this study provides preclinical evidence that UA has the potential to serve as a novel therapeutic agent for enhancing the sensitivity of PDAC to immunotherapy, specifically ICB.

In sum, the investigations by Wang et al. and Mehra et al. underscore the significant therapeutic potential of UB and UA in the context of CRC and PDAC, respectively. Wang et al. demonstrated that UB not only prevents the development of CRC but also modifies the gut microbiota and enhances the immunological environment of tumors, leading to reduced PD-L1 expression and improved outcomes when combined with traditional therapies. Their findings suggest that UB could serve as both a preventive and adjunctive treatment strategy, potentially improving the prognosis for CRC patients and paving the way for innovative therapeutic approaches. Similarly, Mehra et al. highlighted UA’s capacity to restructure the TME in PDAC, enhancing T cell infiltration and reducing markers of immune exhaustion. By demonstrating that UA can improve the efficacy of ICB therapy, they provide compelling evidence for UA as a novel therapeutic agent that could significantly sensitize PDAC to immunotherapy, potentially extending patient survival. Together, these studies illuminate the vital roles that metabolites derived from gut microbiota can play in cancer therapy. By harnessing the immunomodulatory effects of UB and UA, there is potential for the development of new, more effective treatment strategies that not only target tumor cells but also enhance the body’s own immune response. Future research should focus on clinical validation of these findings, exploring optimal dosing regimens and combinations with existing therapies to maximize therapeutic benefits and improve patient outcomes in CRC and PDAC.

## 4 Another microbial-derived metabolite

### 4.1 Microbial-derived-formate

Ternes et al. delved into the intricate interplay between the gut microbiome and CRC, shedding light on its capacity to modulate the immune system and foster tumor growth (241). The findings underscore that various gut-derived bacteria possess the potential to fuel tumor formation, underscoring the microbiome’s pivotal role in CRC development (241). However, the study underscores a significant gap in comprehending the interaction between the gut microbiota and the host, particularly concerning the metabolic processes of tumor cells.

Previous metagenomics research has identified bacterial formate oxidation as a prevalent metabolic pathway in a chemically induced colitis animal model (309). Formate, primarily sourced from the gut microbiome, is undetectable in animals lacking gut bacteria and is synthesized and excreted by diverse bacteria (310, 311). SPF mice exhibited an imbalance in the gut microbiota, leading to an elevation in formate concentration in the intestinal lumen, reaching millimolar values (309). These metabolic shifts may potentially facilitate the establishment and fitness benefits of opportunistic pathogens, including formate dehydrogenase and terminal oxidase genes in mouse colitis models (309). The study primarily focuses on formate, a metabolite produced by *Fusobacterium nucleatum*, a bacterium associated with CRC (241). The research verifies that formate plays a role in promoting CRC development and identifies molecular patterns linking different CRC types with Fusobacterium presence. Co-culturing *Fusobacterium nucleatum* with CRC cells from patients promotes tumor growth, accompanied by significant metabolic alterations, including increased formate secretion and changes in glutamine processing by cancer cells (241). Evidence indicates that AhR activation during murine Th17 cell development augments the numbers of Th17 T cells and their cytokine production (312). The study elucidates the mechanism by which microbiota-generated formate contributes to CRC tumor invasion. This involves the activation of AhR signaling, concurrently enhancing cancer stem cell characteristics (241). Empirical data from mouse studies demonstrate that *Fusobacterium nucleatum* or formate administration leads to a higher tumor occurrence or larger size, accompanied by an increase in proinflammatory Th17 cells (241). Importantly, the study transcends mere observational findings and identifies formate as a gut-derived oncometabolite with relevance to CRC progression. This characterization positions formate as a significant factor in the intricate interplay between the gut microbiome and CRC. The comprehensive findings contribute valuable insights into the molecular and metabolic aspects of CRC development, paving the way for a deeper understanding of potential therapeutic interventions or preventive strategies targeting the gut microbiome and its metabolites in CRC.

### 4.2 Microbial-derived-desaminotyrosine

Joachim et al. investigated the effect of the microbial metabolite DAT on ICI-enhanced cancer immunotherapy and T-cell priming (29). The researchers address the issue of individual variations in ICI response during cancer treatment, specifically investigating the correlation between the composition of the gut microbiota and the diverse outcomes of ICI therapy. Utilizing a live animal model with C57Bl/6j mice, the team administers the bacterial-derived metabolite DAT to modulate IFN-I and improve ICI treatment (29).

Joachim et al. demonstrate that administering DAT to mice leads to a deceleration in tumor development and amplifies the efficacy of ICI immunotherapy, particularly when combined with anti-CTLA-4 or anti-PD-1 treatments (29). Moreover, they establish that DAT’s enhancement of antitumor immunity is associated with heightened activation of T cells and natural killer cells within the TME (29). The researchers intentionally disrupt the balance of gut microbes using broad-spectrum antibiotics to simulate resistance to ICI. The presence of IFN-I is pivotal for the immune system’s detection and elimination of cancer cells (313–315). Existing research indicates that the effectiveness of ICI therapy against malignant melanoma is completely nullified in mice with a hereditary deficiency in IFN-I signaling (316). However, the specific triggers responsible for initiating IFN-I signaling in this context remain unidentified. Microbial metabolites, such as DAT, have demonstrated protective effects against influenza or SARS−CoV−2 infection by augmenting IFN-I signaling (317).

The recent investigation reveals that the enhanced immune response to tumors with DAT hinges on the activation of host IFN-I signaling, indicating the existence of a specific molecular mechanism that underlies the observed benefits (29). Additionally, DAT significantly augments the proliferation of antigen-specific T lymphocytes following vaccination with an adjuvant that induces IFN-I (29). Notably, DAT supplementation counteracts the adverse effects of dysbiosis induced by broad-spectrum antibiotics on the immune response to tumors mediated by anti-CTLA-4 treatment (29). Oral administration of DAT induces changes in the composition of the gut microbiota, leading to an increased abundance of bacterial taxa associated with a positive response to ICI immunotherapy (29). In conclusion, the study introduces a promising therapeutic approach using DAT, a bacterial-derived metabolite, to overcome resistance to ICI, especially in patients with a history of broad-spectrum antibiotic use and associated loss of gut microbial diversity. The findings contribute valuable insights into the potential molecular mechanisms involved in enhancing the antitumor immune response and offer a novel strategy for improving cancer immunotherapy outcomes.

### 4.3 Microbial-derived succinic acid

Jiang et al. conducted an investigation into the impact of succinic acid produced by *Fusobacterium nucleatum* on the development of resistance to immunotherapy in CRC (30). The primary objective was to unravel the mechanisms that render some CRC patients insensitive to ICB treatment with anti-PD-1. *Fusobacterium nucleatum*, a detrimental bacterium found in the intestines, is associated with an unfavorable prognosis for CRC patients (318). Metagenomic analyses have unveiled several metabolites generated by *Fusobacterium nucleatum*, including succinic acid, formate, and propionate (241). Specifically, it has been revealed that *Fusobacterium nucleatum* promotes the stemness, invasion, and metastasis of CRC cells through its metabolite formate (241). In a recent study, Jiang et al. demonstrated that patients with metastatic CRC who did not respond to immunotherapy exhibited a higher prevalence of *Fusobacterium nucleatum* and increased concentrations of succinic acid (30). Furthermore, their findings indicated that transferring fecal microbiota from individuals who positively responded to anti-PD-1 treatment and had low levels of *Fusobacterium nucleatum* led to increased sensitivity to the treatment in mice. Conversely, transferring fecal microbiota from individuals who did not respond to the treatment and had high levels of *Fusobacterium nucleatum* did not confer sensitivity to the treatment in mice. These results highlight a clear connection between the quantity of *Fusobacterium nucleatum* and the response to immunotherapy (30). In a recent study, Gao et al. discovered that the efficacy of PD-L1 inhibition in CRC was enhanced by directly injecting *Fusobacterium nucleatum* into the tumor (319). Conversely, Jiang et al. found that intestinal *Fusobacterium nucleatum* diminished the effectiveness of anti-PD-1 through its metabolite succinic acid (30). The influence of *Fusobacterium nucleatum* in diminishing the effectiveness of anti-PD-1 treatment and inhibiting the cGAS pathway is believed to be dependent on the presence of its metabolite, succinic acid. This association has been confirmed through the use of frdA-deficient *Fusobacterium nucleatum* (30). Gao et al. found that direct injection of *Fusobacterium nucleatum* into tumor tissues in mice can effectively establish colonization and enhance the efficacy of PD-L1 inhibition. Additionally, bacterial infection can activate NF-kB (p65) to increase the expression of PD-L1. Their research revealed that succinic acid produced by *Fusobacterium nucleatum* plays a crucial role in inhibiting the cGAS-interferon-b pathway (30). This inhibition resulted in a decrease in the immune system’s capacity to combat tumors by restricting the movement of CD8+ T cells to the TME in living organisms. The current findings indicate that the effectiveness of immune ICB treatment in subcutaneous tumor models relies on antitumor immunity. This is supported by the fact that tumor development was not stimulated, and the immune response was not suppressed when *Fusobacterium nucleatum* was introduced without the presence of anti-PD1 (30). This result aligns with the outcomes documented in previous research on other microorganisms associated with ICB. Furthermore, they demonstrated that administering the antibiotic metronidazole effectively reduced the presence of *Fusobacterium nucleatum* in the intestines, leading to a decrease in succinic acid levels in the bloodstream. Consequently, this restoration reinstated the sensitivity of malignancies to immunotherapy *in vivo* (30). The results provide insight into the interaction of the gut microbiota, specific metabolites (succinic acid), and the immune system in relation to CRC and immunotherapy. The findings from Jiang et al. have significant implications for the future of immunotherapy in CRC. By elucidating the role of Fusobacterium nucleatum and its metabolite succinic acid in mediating resistance to anti-PD-1 treatment, this study highlights the potential for microbiome-targeted therapies to enhance immunotherapy efficacy. Specifically, the identification of the cGAS-interferon-β pathway as a critical mechanism of resistance opens new avenues for developing interventions that could restore sensitivity to immunotherapy in CRC patients characterized by high levels of Fusobacterium nucleatum.

## 5 Challenges and future directions

The exploration of microbial-derived metabolites for their potential applications in cancer immunity, immunotherapy, and chemotherapy has opened up new avenues in cancer research. However, this promising field is not without its challenges. Understanding and overcoming these obstacles are crucial for the successful development of novel therapeutic strategies. Despite extensive efforts to improve therapeutic efficacy and mitigate detrimental toxicity, the primary challenges in managing cancer therapy revolve around therapeutic resistance and adverse effects (320). Currently, the absence of a standardized methodology, encompassing variations in sample selection and collection, technology, data quality, and resource analysis, hinders the assurance of homogeneity and consistency in understanding the microbial influence on cancer mechanisms. Divergent sampling from identical patients may yield highly disparate outcomes. For instance, while the composition and abundance of microorganisms inhabiting the mucosa of the digestive system are comparable to those found in feces, they are not precisely identical (321). An impediment in employing multi-omic techniques lies in the need to develop tools for identifying the most suitable metabolites and bacteria for further investigation. Optimal conclusions can be drawn by integrating and concurrently analyzing findings from metagenomic/metabolomic or metagenomic/metatranscriptomic datasets. This approach allows the overlay and anticipation of the metabolites correlated to a bacterial gene/gene transcript, thereby enhancing the capacity to focus on a dynamic sequence of occurrences (322). These capabilities are currently in their nascent stages, with anticipated advancements in data processing in the near future. Moreover, the detection of specific compositional and functional microbial fingerprints involves technological factors such as sample management, DNA extraction, bioinformatics, and data collection. Some individuals may opt for 16 S rRNA sequencing of saliva or bile samples, while others may analyze stool samples. High-throughput data can be generated through either next-generation sequencing (NGS) or third-generation sequencing (Nanopore or SMRT sequencing), contributing to the diversity of data resources but also presenting challenges in terms of accessibility. To address these challenges, implementing a comprehensive “standard operating procedure (SOP)” for all methodologies in the future could be beneficial. However, achieving consensus on sample collection, technique selection, and data sharing and analysis would be necessary, despite the various protocols already proposed by the Microbiome Quality Control (MBQC) project consortium (323).

Moreover, the application of microbial techniques may encounter challenges due to individual variations in biology, in addition to the methodological obstacles mentioned earlier. Various factors, such as genetics, dietary habits, age, gender, coexisting medical conditions, and geographical differences, can influence the characteristics of the human microbiota (324, 325). Human microbiomes exhibit considerable variability among individuals, influencing the composition and functionality of microbial-derived metabolites. Tailoring therapies to account for this inter-individual variability poses a substantial challenge in achieving consistent and effective treatment outcomes. Despite the growing recognition of the impact of microbial-derived metabolites on cancer immunity, there is still a limited understanding of the underlying mechanisms governing their interactions with the immune system. Unraveling these complex pathways is essential for designing targeted and efficient therapeutic interventions. The safety profile of microbial-derived metabolites must be thoroughly assessed to mitigate potential adverse effects. Ensuring that these metabolites selectively target cancer cells without harming healthy tissues is a critical aspect of developing safe and well-tolerated therapies. While challenges exist, the future holds great promise for the integration of microbial-derived metabolites into cancer treatment paradigms. Several key directions merit exploration:

- Advancements in precision medicine can contribute to tailoring microbial-derived metabolite therapies based on individualized patient profiles. This includes leveraging genomic and metagenomic data to identify specific microbial strains and metabolites that would be most beneficial for a given patient.
- Integrating systems biology and multi-omics approaches can provide a comprehensive understanding of the complex interplay between microbial metabolites, host immune responses, and cancer development. This holistic perspective will enhance our ability to identify novel therapeutic targets and optimize treatment strategies.
- Harnessing synthetic biology techniques enables the engineering of microbial strains to produce metabolites with enhanced anti-cancer properties. This approach holds the potential to design tailor-made metabolites that address specific challenges associated with cancer immunity and therapy.
- Rigorous clinical trials are essential for validating the safety and efficacy of microbial-derived metabolites in diverse cancer populations. The translation of preclinical findings into clinically applicable therapies is a crucial step toward establishing these metabolites as integral components of cancer treatment regimens.

In conclusion, addressing the challenges and exploring future directions in harnessing microbial-derived metabolites for cancer immunity, immunotherapy, and chemotherapy requires interdisciplinary collaboration, technological innovation, and a deep commitment to advancing our understanding of the intricate relationships between the microbiome and cancer. With continued research and development, microbial-derived metabolites may emerge as powerful tools in the fight against cancer, offering novel and targeted therapeutic options for patients.

## 6 Conclusion

In this review we provided a comprehensive exploration of the intricate interplay between microbial-derived metabolites and their pivotal role as regulators of anti-tumor immunity, immunotherapy, and chemotherapy. The intricate mechanisms through which these metabolic mediators influence various facets of the immune response against tumors have been elucidated, shedding light on novel therapeutic avenues in cancer treatment. The findings underscore the significance of specific metabolites, such as short-chain fatty acids, tryptophan metabolites, and others, in influencing immune responses and therapeutic outcomes. The impact of gut microbiota on the efficacy of immunotherapy and chemotherapy has been a focal point, emphasizing the need for a deeper understanding of these interactions. As we look towards the future, it is evident that advancements in data processing, standardization of methodologies, and technological innovations will further refine our understanding of microbial-derived metabolites in cancer therapeutics. The identification of key metabolites and bacteria through multi-omic approaches holds promise for developing targeted interventions. The prospect of establishing a comprehensive “standard operating procedure” for methodologies, as suggested by the Microbiome Quality Control project consortium, could contribute to overcoming challenges related to sample collection, technique selection, and data analysis.

However, it is crucial to acknowledge the existing challenges, including individual biological variations and the diverse methodologies employed in sample collection and sequencing technologies. Future research endeavors should focus on harmonizing protocols, considering biological variables, and addressing methodological inconsistencies to ensure the reliability and reproducibility of results. In the realm of cancer therapy, the role of microbial-derived metabolites opens avenues for personalized and precision medicine. Harnessing the potential of these metabolites may lead to the development of innovative therapeutic strategies, enhancing the effectiveness of existing treatments and potentially mitigating resistance issues. In summary, this review not only consolidates our current understanding of microbial-derived metabolites in anti-tumor immunity and cancer therapy but also highlights the exciting prospects and challenges that lie ahead. The continued exploration of these metabolic mediators is poised to revolutionize cancer treatment strategies, paving the way for more targeted, efficient, and personalized therapeutic interventions.
